# Subcellular localization of PD‐L1 and cell‐cycle‐dependent expression of nuclear PD‐L1 variants: implications for head and neck cancer cell functions and therapeutic efficacy

**DOI:** 10.1002/1878-0261.13567

**Published:** 2023-12-26

**Authors:** Daniela Schulz, Laura Feulner, Dominique Santos Rubenich, Sina Heimer, Sophia Rohrmüller, Yvonne Reinders, Marcelo Falchetti, Martin Wetzel, Elizandra Braganhol, Edroaldo Lummertz da Rocha, Nicole Schäfer, Sabine Stöckl, Gero Brockhoff, Anja K. Wege, Jürgen Fritsch, Fabian Pohl, Torsten E. Reichert, Tobias Ettl, Richard J. Bauer

**Affiliations:** ^1^ Department of Oral and Maxillofacial Surgery University Hospital Regensburg Germany; ^2^ Department of Oral and Maxillofacial Surgery, Experimental Oral and Maxillofacial Surgery, Center for Medical Biotechnology University Hospital Regensburg Germany; ^3^ Postgraduation program in Biosciences Federal University of Health Sciences from Porto Alegre Brazil; ^4^ Leibniz‐Institute for Analytical Sciences, ISAS e.V. Dortmund Germany; ^5^ Department of Microbiology, Immunology and Parasitology Federal University of Santa Catarina Florianópolis Brazil; ^6^ Department of Basic Health Sciences Federal University of Health Sciences from Porto Alegre Brazil; ^7^ Department of Orthopaedic Surgery, Experimental Orthopaedics University of Regensburg Germany; ^8^ Department of Orthopaedic Surgery, Experimental Orthopaedics, Center for Medical Biotechnology University Hospital Regensburg Germany; ^9^ Department of Gynecology and Obstetrics University Medical Center Regensburg Germany; ^10^ Department of Infection Prevention and Infectious Diseases University Medical Center Regensburg Germany; ^11^ Department of Radiotherapy University Medical Center Regensburg Germany

**Keywords:** DNA remodeling, HNSCC, nPD‐L1, nuclear trafficking, subcellular protein fractionation, vimentin immunotherapy

## Abstract

The programmed cell death 1 ligand 1 (PD‐L1)/programmed cell death protein 1 (PD‐1) axis is primarily associated with immunosuppression in cytotoxic T lymphocytes (CTLs). However, mounting evidence is supporting the thesis that PD‐L1 not only functions as a ligand but mediates additional cellular functions in tumor cells. Moreover, it has been demonstrated that PD‐L1 is not exclusively localized at the cellular membrane. Subcellular fractionation revealed the presence of PD‐L1 in various cellular compartments of six well‐characterized head and neck cancer (HNC) cell lines, including the nucleus. Via Western blotting, we detected PD‐L1 in its well‐known glycosylated/deglycosylated state at 40–55 kDa. In addition, we detected previously unknown PD‐L1 variants with a molecular weight at approximately 70 and > 150 kDa exclusively in nuclear protein fractions. These *in vitro* findings were confirmed with primary tumor samples from head and neck squamous cell carcinoma (HNSCC) patients. Furthermore, we demonstrated that nuclear PD‐L1 variant expression is cell‐cycle‐dependent. Immunofluorescence staining of PD‐L1 in different cell cycle phases of synchronized HNC cells supported these observations. Mechanisms of nuclear PD‐L1 trafficking remain less understood; however, proximity ligation assays showed a cell‐cycle‐dependent interaction of the cytoskeletal protein vimentin with PD‐L1, whereas vimentin could serve as a potential shuttle for nuclear PD‐L1 transportation. Mass spectrometry after PD‐L1 co‐immunoprecipitation, followed by gene ontology analysis, indicated interaction of nuclear PD‐L1 with proteins involved in DNA remodeling and messenger RNA (mRNA) splicing. Our results in HNC cells suggest a highly complex regulation of PD‐L1 and multiple tumor cell‐intrinsic functions, independent of immune regulation. These observations bear significant implications for the therapeutic efficacy of immune checkpoint inhibition.

## Graphical abstract

Both the membrane and nucleus of head and neck cancer cells express PD‐L1. PD‐L1 is translocated from the membrane into the nucleus via vimentin. We detected high‐molecular‐weight PD‐L1 variants in the nucleus. Their expression is cell‐cycle‐dependent and involves interaction with proteins related to DNA remodeling and mRNA splicing. Multimerization likely causes PD‐L1's high weight in cancer patient tumors.

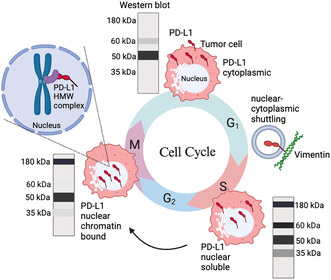

**Abbreviations**
2‐ME: β‐mercaptoethanol
APCs: antigen‐presenting cells
BCA: bicinchoninic acid
BSA: bovine serum albumin
CDK4/6: cyclin‐dependent kinase 4/6
CoIP: co‐immunoprecipitation
CP: cytoplasmic
cPD‐L1: cytoplasmic PD‐L1
CPS: combined positive score
CS: cytoskeletal
CST: Cell Signaling Technologies
CSTF1: cleavage stimulation factor subunit 1
CTCF: corrected total cell fluorescence
CTLs: cytotoxic T lymphocytes
d: day
DMSO: dimethyl sulfoxide
DNA: deoxyribonucleic acid
EGF: epidermal growth factor
Endo H: endoglycosidase H
ER: endoplasmic reticulum
EV PD‐L1: extracellular vesicle PD‐L1
Gy: Gray
HB‐V: homogenization buffer‐Vladimir
HDAC2: histone deacetylase 2
HNC: head and neck cancer
HNRNPH2: heterogeneous nuclear ribonucleoprotein H2
HNSCC: head and neck squamous cell carcinoma
IFN‐γ: interferon gamma
IgG: immunoglobulin G
IRR: irradiation
KD: knockdown
kDa: kilodalton
LC–MS: liquid chromatography – mass Spectrometry
mAB: monoclonal antibody
MACROH2A1: macroH2A.1 histone
mPD‐L1: membrane PD‐L1
mRNA: messenger RNA
NCB: nuclear chromatin‐bound
NONO: non‐POU domain containing octamer binding
NP: nucleus depletionn
NS: nuclear soluble
NT: non‐targeting control
pAb: polyclonal antibody
PBS: phosphate buffered saline
PD‐1: programmed cell death protein 1
PD‐L1: nuclear PD‐L1
PD‐L1: programmed cell death 1 ligand 1
PLA: proximiy ligation assay
PNGase F: *N*‐glycosidase F
PTM: post‐translational modification
rcf: relative centrifugal force
RIPA: radio‐immunoprecipitation buffer
RNA: ribonucleic acid
RSLC: rapid separation liquid chromatography
RT: room temperature
SDS/PAGE: sodium dodecyl sulfate – polyacrylamide gel electrophoresis
sPD‐L1: soluble PD‐L1F
SPR: S phase release
TBS: Tris‐buffered saline
WAPL: Wing Apart‐Like

## 1 Introduction

Head and neck squamous cell carcinoma (HNSCC) is the 8th most prevalent cancer worldwide. Annually, there are nearly 900 000 new diagnoses, with increasing incidence over the past 10 years [1]. The standard therapeutic treatment for HNSCC involves a multimodal therapy of surgery, chemo‐ and radiotherapy. Despite significant advances in medical research, the 5‐year overall survival rate for HNSCC patients has not shown significant improvement over the past few decades and is still around 50% [2]. Early clinical trials in patients with recurrent or metastatic HNSCC, for whom the chances of improvement after platinum‐based chemotherapy have been poor, initially showed impressive clinical results using the anti‐PD‐1 antibodies nivolumab or pembrolizumab [3, 4]. The PD‐1/PD‐L1 axis plays a critical role in regulating T cell activity [5]. The receptor PD‐1 binds to membrane‐bound ligand PD‐L1 and thereby attenuates cytotoxic T cell (CTLs) activity [6]. PD‐L1 is expressed in many different cell types, such as T and B lymphocytes, monocytes, APCs, and epithelial cells [7]. Furthermore, PD‐L1 expression is upregulated in response to EGF and proinflammatory cytokines, such as IFN‐γ. Various cancer entities exploit overexpression of co‐inhibitory ligands to evade the immune attack of CTLs. This immune escape mechanism is observed in many solid tumors, such as colorectal, gastric, lung, and esophageal cancer, hepatocellular carcinoma, melanoma, glioblastoma, and squamous cell carcinoma of the oral cavity [8, 9, 10, 11].

Despite some promising outcomes, antibody‐based PD‐1/PD‐L1 immunotherapy in HNSCC often yields limited improvement or even hyperprogression. Underlying mechanisms have not yet been fully understood [12, 13]. The heterogeneity of PD‐L1 expression and the lack of reliable biomarkers still leads to unpredictable outcomes [14]. To date, most studies have focused on the immunogenic function of the PD‐1/PD‐L1 interaction. However, there is increasing evidence that PD‐L1 also mediates cell‐intrinsic functions that may trigger tumor‐promoting effects [15, 16, 17, 18]. In previous studies, also we have demonstrated the strong influence of PD‐L1 on cell cycle progression and proliferation, migration and invasion, as well as survival after irradiation [19, 20, 21]. The expression level of PD‐L1 was depending on the differentiation status of the cell. Even more interestingly, the cellular localization of PD‐L1, including PD‐L1 membrane expression, changed in response to treatment. Both, cell cycle inhibition and irradiation, lead to a differential cellular translocation of PD‐L1 [21]. This observed effect could reduce the efficacy of immunotherapy and promote tumor cell resistance [22]. Recent studies have shown a correlation between PD‐L1 expression in tumor cells and the response to PD‐1/PD‐L1 blockade [23, 24], while also the specific cellular localization of PD‐L1 may play an important role. Determining the efficacy of antibody‐based immunotherapy relies on identifying patients who benefit from PD‐1/PD‐L1 therapy. For HNC it is the combined positive score (CPS). However, only membrane expression of PD‐L1 is considered relevant for scoring [25]. Nevertheless, recent research suggests that the precise cellular localization of PD‐L1 may play a crucial role in the success of antibody‐based immunotherapy. While tumor cells can exhibit PD‐L1 overexpression, its intracellular (trans‐)location can lead to less effective immunotherapy and potentially foster tumor‐promoting characteristics. Therefore, understanding the intracellular presence of PD‐L1, rather than just its overexpression, is crucial to predict treatment response. Currently, monitoring changes in PD‐L1 status during therapy is lacking. Moreover, PD‐1/PD‐L1 therapy currently is only approved for patients in advanced stages of recurrent and metastatic HNC. Predicting efficacy of antibody‐based immunotherapy would be highly relevant for patients [26].

In this study, we performed subcellular protein fractionation of HNC cells and discovered that PD‐L1 expression is not limited to the cellular membrane but is also present in the cytoplasmic and nuclear fractions. We identified novel PD‐L1 variants of high molecular weight within the soluble nuclear fraction and the chromatin‐bound protein fraction. The localization and expression of PD‐L1 were found to be dependent on the phase of the cell cycle. We also observed a cell cycle‐dependent interaction between PD‐L1 and vimentin, which potentially facilitates the nuclear transport of PD‐L1. Inside the nucleus, PD‐L1 interacts with proteins involved in mRNA splicing and DNA remodeling. Recently, there have been some new publications investigating the translocation of PD‐L1 into the nucleus [22, 27, 28], where it appears to be involved in sister chromatid segregation [28]. Deacetylation of PD‐L1 on the plasma membrane enables its binding with HIP1R and AP2B1 for endocytosis. PD‐L1 also interacts with vimentin to traffic via the cytoskeleton and enters the nucleus through Importin‐α1. Furthermore, a study by Gao et al. identified that HDAC2 deacetylase interacts with PD‐L1 and reduces p300‐mediated acetylation of PD‐L1. Therefore, combining a HDAC2 inhibitor with PD‐1/PD‐L1 blockade could be a novel synergistic strategy for cancer immunotherapy [27]. Moreover, Zhang et al. [29] observed a correlation between the expression of PD‐L1 and the cell cycle phase. In their study, inhibition of CDK4/6 affected PD‐L1 expression in mouse embryonic fibroblasts, and knockout of cyclin D1 resulted in increased PD‐L1 levels. Cell cycle‐regulated expression of PD‐L1 indicates their potential involvement in cell cycle‐related processes.

Our findings provide valuable insights into the heterogeneous responses observed with PD‐1/PD‐L1 antibody therapy for the treatment of HNC. A deeper understanding of the cellular localization and expression patterns of PD‐L1 in different cellular compartments during cell cycle progression will help to optimize synergistic treatment modalities to further improve antibody‐based immunotherapy.

## 2 Material and methods

### 2.1 Cell lines and culture conditions

The human head and neck cancer cell lines A‐253 (RRID: CVCL_1060), Detroit 562 (D‐562, RRID: CVCL_1171), FaDu (RRID: CVCL_1218), SCC‐9 (RRID: CVCL_1685), and SCC‐15 (RRID: CVCL_1681) were ordered from ATCC (Manassas, VA, USA) as Head and Neck Panel TCP‐1012. The head and neck cancer cell line PCI 52 (RRID: CVCL_RJ99) was kindly provided by Prof. Dr. Theresa. L. Whiteside [University of Pittsburgh Cancer Institute (PCI), Pittsburgh, PA, USA] in 2013 [30]. HNC cell lines were maintained in DMEM (PanBiotech, Aidenbach, Germany) supplemented with 10% fetal calf serum (FCS, Gibco, Carlsbad, CA, USA), 1% l‐glutamine (Sigma‐Aldrich, St. Louis, MO, USA) and 1% penicillin/streptomycin (Sigma‐Aldrich) at 37 °C in a 5% CO_2_ humidified atmosphere. The medium was changed every 2–3 days, and the cells were passaged prior reaching confluence. Detachment of adherent cells was achieved by incubation with 0.05% Accutase solution (Merck, Darmstadt, Germany) for 5–10 min at 37 °C. Patient samples were provided from HNSCC patients from the Department of Oral and Maxillofacial surgery, University Hospital Regensburg, and processed immediately after resection. All cell lines are regularly tested for mycoplasma. The used cell lines were free of mycoplasma.

### 2.2 Primary HNSCC tissue

Patient recruitment primary HNSCC tissue samples was conducted at the Department of Oral and Maxillofacial Surgery. All participants involved in this study were thoroughly informed about the nature and purpose of the study, as well as the procedures involved. Written informed consent was obtained from all individual participants involved in the study prior to sample collection. Ethical approval for this study was granted by the Ethics Committee at University Hospital Regensburg (AZ:16‐299‐101). All procedures performed in this study involving human participants were conducted in compliance with the ethical standards of the institutional research committee and with the 1964 Helsinki Declaration and its later amendments or comparable ethical standards.

### 2.3 Cell authentication

Cell line authentication of the human HNC cell line PCI 52 was performed by the Leibniz Institute German Collection of Microorganisms and Cell Cultures (DSMZ, Berlin, Germany) via STR‐DNA‐typing using nonaplex PCR [A1806475‐1].

### 2.4 Cell cycle inhibition

Palbociclib (PD 0332991 isethionate), a potent cyclin‐dependent CDK4/6 inhibitor inducing G1 cell cycle arrest, was obtained from Tocris Bioscience (Bristol, UK). A 10 mm stock solution was prepared in dimethyl sulfoxide (DMSO) and stored in aliquots at −20 °C.

Aphidicolin, a specific inhibitor of DNA polymerase α and δ in eukaryotic cells that blocks the cell cycle at early S phase, was obtained from Merck. A 2.9 mm stock solution was prepared in DMSO and stored in aliquots at −20 °C.

Nocodazole, which interferes with the structure and function of microtubules in interphase of mitotic cells blocking cell cycle in G2/M phase, was obtained from Sigma‐Aldrich as 5 mg·mL^−1^ ready made DMSO solution, stored in aliquots at −20 °C.

Prior to application, dilutions of cell cycle inhibitors were prepared, using antibiotics‐free medium. Treatment with cell cycle inhibitors was not started earlier than 24 h (h) after seeding. As a control, antibiotics‐free medium containing DMSO was used, which corresponded to the DMSO concentration in the highest dose of the cell cycle inhibitor. This method has already been described in [21].

### 2.5 Irradiation

External irradiation was delivered through an anterior portal by a 6‐MV linear accelerator, which emits a photon beam for a total irradiation dose of 8 Gy (3 Gy·min^−1^; Primus, Siemens, Nurnberg, Germany) at room temperature. Irradiation was not performed earlier than 24 h after seeding. Cell culture plates were located on the acceleration treatment couch. Due to the build‐up effect, 2 cm thick Plexiglas plates were positioned above and below the tissue culture vials. Non‐irradiated cells served as control. This method has also been described in [31].

### 2.6 Flow cytometry

The inhibitory concentrations of the cell cycle inhibitors were adjusted using cell cycle analysis and apoptosis experiments via flow cytometry. Experiments were performed at the FACS Canto II (BD Biosciences, Franklin Lakes, NJ, USA) cytometer with facsdiva software 7.0 (BD Biosciences).

For S phase analysis, 5 × 10^5^ cells in suspension were washed and fixated with 500 μL of 70% methanol (Carl Roth, Karlsruhe, Germany) overnight at 4 °C. RNA digestion was conducted with 25 μg Ribonuclease A (Sigma‐Aldrich) for 20 min at 37 °C. DAPI staining was as performed with 250 ng DAPI (Sigma‐Aldrich) for at least 30 min at room temperature (RT). Signal intensity was measured with the cytometer FACS Canto II (BD Biosciences), facsdiva software 7.0 (BD Biosciences) and analyzed with ModFit LT software 5.0 (Verity Software House, Topsham, ME, USA).

Cell viability was analyzed via flow cytometry. All incubation steps were performed at 4 °C or on ice. Cell suspensions with 5 × 10^5^ cells were washed with PBS (Sigma‐Aldrich). Annexin V‐FITC (Immunotools GmbH, Friesoythe, Germany) staining was performed according to the manufacturer's protocol. Prior to the measurement, 50 ng DAPI (Sigma‐Aldrich) was added to the cells and incubated for 1 min. Dot plots were created with Flowing Software 2.5.1 (Turku Centre for Biotechnol., Uni. Turku, Finland).

### 2.7 siRNA knockdown (KD)

To decrease PD‐L1 expression (KD), cells were transiently transfected with small interfering RNA (siRNA) directed against the human PD‐L1 gene CD274. Dharmafect‐1 (Thermo Scientific, Waltham, MA, USA) was used as transfection reagent according to the manufacturer's instructions. Transfection was performed in 6‐well plates (Corning, NY, USA). A cell suspension of 2 × 10^5^ cells was added to prepared transfection complexes. Transfection with 25 nm siRNA was carried out in DMEM growth medium without antibiotics for 72 h. The siGENOME Human CD274 siRNA SMARTpool (M‐015836‐01‐0005, Horizon Discovery, Cambridge, UK) consists of four siRNA target sequences. This method has already been described in [21]. The ON‐TARGETplus Non‐Targeting Control Pool (D‐001810‐10‐20, Horizon Discovery) was used as control. The siRNA sequences used for PD‐L1 KD experiments are listed in Table 1.

**Table 1:** siRNA sequences used for PD‐L1 KD experiments.

PD‐L1 siRNA SMARTpool	Non‐targeting control pool
UGAAAGGACUCACUUGGUA (D‐015836‐01)	UGGUUUACAUGUCGACUAA (D‐001810‐01)
CAUAGUAGCUACAGACAGA (D‐015836‐02)	UGGUUUACAUGUUGUGUGA (D‐001810‐02)
AGACCUGGCUGCACUAAUU (D‐015836‐03)	UGGUUUACAUGUUUUCUGA (D‐001810‐03)
GGACCUAUAUGUGGUAGAG (D‐015836‐04)	UGGUUUACAUGUUUUCCUA (D‐001810‐04)

### 2.8 Nucleus depletion

For depletion of nuclei ice‐cold HB‐V PIC buffer [250 mm sucrose, 15 mm HEPEs, 0.5 mm MgCl_2_ at pH 7.4 including Protease Inhibitor Cocktail (Roche, Penzberg, Germany)] was added to cells. Cells were homogenized with ultrasound and centrifuged at 750 relative centrifugal force (rcf) for 4 min, duration of ultrasound exposure was adjusted after visual inspection of the supernatant. The supernatant was collected. HB‐V‐PIC was added to the remaining cells for further homogenization, four times in total. The post nuclear supernatant was used for the WB analysis. This protocol is described in Fritsch et al. as part of the immunomagnetic purification of signaling organelles [32].

### 2.9 PNGase F and Endo H digestion

*N*‐glycosidase F (PNGase F) from Promega (Fitchburg, WI, USA) was employed to catalyze the cleavage of N‐linked oligosaccharides following the manufacturer's denaturing conditions protocol for SDS/PAGE. Each digestion utilized 20 μg of protein, with an incubation period of 3 h at 37 °C.

Endoglycosidase H (Endo H) from Promega was employed to enzymatically cleave the diacetylchitobiose core of oligosaccharides, specifically between the two *N*‐acetylglucosamine residues, while leaving one *N*‐acetylglucosamine residue attached to the asparagine, following the manufacturer's protocol. It is important to note that this differs from PNGase F, which cleaves all asparagine‐linked oligosaccharides except those containing fucose. Each digestion utilized 20 μg of protein and involved an 18 h incubation at 37 °C.

### 2.10 Total protein isolation

For total protein lysates, adherent cells were washed with PBS and directly lysed in radio‐immunoprecipitation buffer (RIPA) (Sigma‐Aldrich).

For total protein lysates of primary tumors, the obtained tissue was washed gently with ice‐cold PBS. Excess liquid was removed. The tissue was cut into small pieces and placed in a pre‐chilled tube for homogenization with a pestle (Kisker Biotech GmbH & Co. KG, Steinfurt, Germany) in RIPA buffer.

To prevent protein degradation, the lysis buffer was complemented with protease inhibitors (cOmplete™ Mini Protease Inhibitor Cocktail) (Roche Diagnostics, Basel, Switzerland). All incubation steps were performed at 4 °C or on ice. To optimize cell lysis, harvested cells, or grinded tissue, was sonicated on ice for 20 s. For removal of cell debris, pellet and supernatant were separated by centrifugation at 16 000 rcf for 10 min at 4 °C. The supernatant was stored at −80 °C for further analysis.

### 2.11 Subcellular protein fractionation

HNC cell lines were fractionated with the Subcellular Protein Fractionation Kit for Cultured Cells (Thermo Scientific) according to the manufacturer's protocol. Cells were harvested with Accutase Solution (Merck). A packed cell volume 50–100 μL was used for each sample. Protease inhibitors (Halt™ Protease Inhibitor Cocktail 100×, Thermo Scientific) were added to each buffer to maintain extract integrity and function. All incubations were performed at 4 °C unless otherwise noted. The first reagent added to a cell pellet caused selective cell membrane permeabilization, releasing soluble cytoplasmic contents. The second reagent dissolved plasma, mitochondria and ER/Golgi membranes without solubilizing nuclear membranes. After recovering the intact nuclei by centrifugation, a third reagent yielded the soluble nuclear extract. A second nuclear extraction with micrococcal nuclease was performed to release chromatin‐bound nuclear proteins. For DNA digestion in the chromatin‐bound nuclear protein fraction, micrococcal nuclease was incubated at 37 °C for 5 min. The recovered insoluble pellet was extracted with the final reagent to isolate cytoskeletal proteins. For long‐term storage, fractions were stored at −80 °C.

Primary tumor cells were fractionated with the Subcellular Protein Fractionation Kit for Tissues (Thermo Scientific) according to the manufacturer's protocol. A tissue weight of 100–200 mg was used for each sample. Primary tumor tissue was washed gently with ice‐cold PBS. Excess liquid was removed. Tissue was cut into small pieces and placed in a pre‐chilled tube for homogenization with a pestle (Kisker Biotech GmbH & Co. KG) in CEB‐buffer. Protease inhibitors (Halt™ Protease Inhibitor Cocktail 100×, Thermo Scientific) were added to each buffer to maintain extract integrity and function. All incubations were performed at 4 °C unless otherwise noted. For the removal of tissue debris, the homogenized tissue was transferred into the Pierce Tissue Strainer (Thermo Scientific) before the second buffer dissolved plasma, mitochondria and ER/Golgi membranes without solubilizing the nuclear membranes. After recovering the intact nuclei by centrifugation, a third buffer yielded the soluble nuclear extract. A second nuclear extraction with micrococcal nuclease was performed to release chromatin‐bound nuclear proteins. For DNA digestion in chromatin‐bound nuclear protein fraction micrococcal nuclease was incubated 37 °C for 15 min. The recovered insoluble pellet is then extracted with the final buffer to isolate cytoskeletal proteins. For long‐term storage, fractions were stored at −80 °C.

As an alternative approach, HNC cell lines were fractionated according to the subcellular fractionation protocol of Abcam (Cambridge, UK) (https://www.abcam.com/protocols/subcellular‐fractionation‐protocol (downloaded on 25.06.2023). Cells were detached mechanically from the cell culture dish by scraping in subcellular fractionation buffer. The nuclear fraction was separated by centrifugation at 720 rcf and purified. The nuclear proteins were resuspended in TBS including 0.1% SDS. The mitochondrial fraction was separated by centrifugation at 10 000 rcf and purified. For the separation of membrane and cytoplasmic proteins, the supernatant was centrifuged at 100 000 rcf (Optima™ MAX‐E Ultracentrifuge, rotor: TLA55, Beckmann Coulter). The supernatant contained the cytoplasmic fraction. The pellet, containing membrane proteins, was purified and dissolved in fractionation buffer. For long‐term storage, fractions were stored at −80 °C.

### 2.12 Western blotting (WB)

For quantitative protein analysis, each sample utilized 20–30 μg of total protein lysate or subcellular protein fraction. Protein concentrations were determined using a bicinchoninic acid (BCA) assay (Merck). The proteins were denatured at 70 °C for 10 min in a Laemmli sample buffer (Bio‐Rad, Hercules, CA, USA) containing 1% β‐mercaptoethanol (2‐ME) (Merck). Samples were separated via SDS/PAGE using either a 10% gel or a precast Mini‐PROTEAN^®^ TGX™ 4–12% gradient Gel (Bio‐Rad) and subsequently transferred onto a PVDF membrane (Roche). The membrane was blocked with either 5% skimmed milk (Carl Roth) or 3% bovine serum albumin (BSA) Fraction V (Biomol, Hamburg, Germany) in a TBS buffer containing 0.1% Tween 20 (Sigma‐Aldrich) for 1 h at RT. Following this, the membrane was incubated overnight at 4 °C with specific primary antibodies. The primary antibodies used for WB analysis included anti‐PD‐L1 (rabbit mAb, #13684, Cell Signaling Technology, Danvers, MA, USA (CST)), anti‐PD‐L1 (rabbit mAb, HRP Conjugate, #51296, CST), anti‐PD‐L2 (rabbit mAb, #83723, CST), anti PD‐1 (rabbit mAb, #86163, CST), anti‐EGF Receptor (rabbit mAb, #4267, CST), anti‐vimentin (rabbit mAb, #5741, CST), anti‐E‐cadherin (mouse mAb, 610182, BD Biosciences), anti‐N‐cadherin (mouse mAb, 610921, BD Biosciences), anti‐Twist (mouse mAb, sc‐81417, Santa Cruz Biotechnology, Dallas, TX, USA), anti‐Snai1 (mouse mAb, sc‐393172, Santa Cruz Biotechnology), and anti‐CD44 (mouse mAb, #3570, CST). For the PD‐L1 CoIP, the anti‐PD‐L1 (rabbit, mAb, HRP Conjugate, #51296, CST) was used as loading control. For signal detection, the membrane was subsequently incubated with horseradish peroxidase (HRP)‐conjugated secondary antibodies. Specifically, goat anti‐rabbit (#32460, Thermo Scientific) and goat anti‐mouse antibodies with stabilized peroxidase conjugation (#32430, Thermo Scientific) were employed. The substrates used were Roti^®^Lumin (Carl Roth) or SuperSignal™ West Femto Maximum Sensitivity Substrate (Thermo Scientific). Colorimetric and chemiluminescent images were processed using the high‐resolution, high‐sensitivity ChemiDoc™ XRS+ Imaging System (Bio‐Rad). Equal loading of proteins was confirmed with Ponceau S (SERVA Electrophoresis GmbH, Heidelberg, Germany) total protein staining, as well as the appropriate housekeeping protein detection. The antibodies used for the loading control and verification of the analyzed subcellular protein fraction were anti‐β‐Actin (rabbit pAb, ab8227, Abcam), anti‐GAPDH (rabbit mAb, #5174, CST), anti‐Calpain 2 Large Subunit (M‐Type) (rabbit pAb, #2539, CST), anti‐Na‐, K‐, ATPase (rabbit, pAb #3010, CST), anti‐EGFR (rabbit, mAb, #4267, CST), anti‐Lamin A/C (rabbit, pAb, #2032, CST), anti‐Histone H3 (1B1B2) (mouse, mAb, #14269, CST), and anti‐β‐Tubulin (rabbit, mAb, #2128, CST). The housekeeping protein was applied to the same membrane following stripping with ReBlot Plus Mild Antibody Stripping Solution (Merck). The analysis was carried out using Image Lab software 6.0.1 (Bio‐Rad). The method was also described in detail in [21].

### 2.13 Co‐immunoprecipitation (CoIP)

For the detection of PD‐L1 interacting partners, each sample utilized 100 μg of protein. To minimize potential non‐specific binding interactions with magnetic beads, all samples underwent pre‐clearance following the manufacturer's guidelines, employing 50 μL of μMACS™ Protein A MicroBeads (Miltenyi Biotec, Bergisch Gladbach, Germany). For CoIP, the pre‐cleared protein lysate was mixed with 50 μg of μMACS™ Protein A MicroBeads (Miltenyi Biotec) and 1 μg of anti‐PD‐L1 (rabbit mAb, clone E1L3N, #13684, CST). This mixture was then incubated on ice for 30 min, in accordance with the manufacturer's instructions. An IgG isotype antibody (rabbit mAb, #3900, CST) served as a control for unspecific capture antibody binding. μ Columns (Miltenyi Biotec) were positioned in the thermoMACS™ Separator (Miltenyi Biotec). After column equilibration with 70% ethanol (Sigma‐Aldrich) and rinsing with RIPA buffer, the mixture was transferred to the columns. Magnetic beads became attached to the columns while non‐bound proteins were eliminated. Proteins bound to the magnetic beads were subsequently eluted using hot (95 °C) Laemmli sample buffer (Bio‐Rad) containing 1% 2‐ME. Following this, LC–MS and SDS/PAGE with WB was conducted.

### 2.14 Immunofluorescent (IF) staining

IF staining was performed in 8‐chamber slides (BD Biosciences). Cells were washed thoroughly with PBS (DPBS, including calcium and magnesium, Gibco) prior to fixation with 4% paraformaldehyde (PFA) for 10 min at RT. Before antibody incubation, cells were washed thoroughly again, and nonspecific binding sites were covered with a blocking solution containing 3% BSA (Biomol) and 0.1% Triton X‐100 (Sigma‐Aldrich). Primary antibody incubation was performed with anti‐PD‐L1 (rabbit mAb, clone E1L3N, #13684, CST) in antibody diluent containing 1% BSA (Biomol) and 0.1% Triton X‐100 (Sigma‐Aldrich) overnight at 4 °C. For signal detection of the target protein host‐specific F(ab′)2‐Goat anti‐Rabbit IgG (H + L), cross‐adsorbed secondary antibody (Thermo Scientific) was incubated for 1 h at 37 °C. Cytoskeleton was stained with Alexa Fluor™ 546 Phalloidin (Thermo Scientific) for 20 min at 37 °C. Slides were mounted with VECTASHIELD Antifade Mounting Medium with DAPI (Biozol Diagnostica Vertrieb GmbH, Eching, Bayern) and covered with a high precision microscope cover glass (Marienfeld Superior, Lauda‐Königshofen, Germany).

### 2.15 Proximity ligation assay (PLA)

PLA was performed in 16‐chamber slides (Lab‐Tek Chamber Slide Glass, Thermo Scientific Nunc) according to the manufacturer's protocol. Cells were fixated with 4% PFA for 10 min at RT and permeabilized with 0.1% Triton X‐100 (Sigma‐Aldrich) for 1 min at RT. Afterwards the cells were incubated with specific antibodies for two different target proteins derived from different hosts, anti‐PD‐L1 (rabbit mAb, clone E1L3N, #13684, CST) and anti‐vimentin (mouse mAb, ab8978, Abcam). The specificity of staining was verified by using isotype controls (rabbit mAb, clone DA1E, IgG XP^®^ Isotype Control #3900, CST and mouse mAb IgG1κ, clone MOPC‐21, Isotype Control, M 5284, Sigma Aldrich) in respective concentrations. As positive controls, anti‐β‐Actin (rabbit pAb, ab8227, Abcam) and anti‐vimentin (mouse mAb, ab8978, Abcam) were selected. For signal detection Duolink^®^ *In Situ* PLA^®^ Probe Anti‐Rabbit PLUS (DUO92002, Sigma‐Aldrich) and Duolink^®^ *In Situ* PLA^®^ Probe Anti‐Mouse MINUS (DUO92004, Sigma‐Aldrich) was used. Additional staining with phalloidin (Alexa Fluor™ 546 Phalloidin, Thermo Scientific) and DAPI (Sigma‐Aldrich) for 45 min at RT in the dark was used for visualization of cytoskeleton and nuclei. Cells were mounted with Dako Fluorescence Mounting Medium (Agilent Technologies Inc., Santa Clara, CA, USA) and coverslipped with a 16‐well coverglass slide (Nalge Nunc International Corporation, Rochester, NY, USA). For signal quantification, single cells were analyzed separately. For every cell, the fluorescence intensity of each PLA signal (red) was measured by fiji imagej software [33]. The corrected total intensity was calculated by subtracting the mean background in relation to the area analyzed (CTCF).

### 2.16 Liquid chromatography − mass spectrometry (LC–MS)

Samples were either processed using a S‐Trap™ (Protifi) procedure according to the manufacturer's instruction or, after separation using 1D‐PAGE equilibrated in 50 mm ammonium bicarbonate buffer, reduced using 10 mm Dithiothreitol (DTT), carbamidoalkylated using incubation in 30 mm iodoacetamide and tryptically digested overnight at 37 °C. The digest is being quenched using 10% trifluoroacetic acid (TFA) and the corresponding peptides were dried. Lyophilized peptides were dissolved in 0.1% (v/v) trifluoroacetic acid (TFA) for subsequent quality control via monolithic HPLC followed by UV‐detection and LC–MS/MS analysis.

All samples were analyzed by nano LC–MS/MS using 1 μg, respectively. Samples were loaded on an UltiMate™ 3000 Rapid Separation Liquid chromatography (RSLC) nanosystem with a ProFlow flow control device coupled to a Q Exactive™ HF Orbitrap™ mass spectrometer (both from Thermo Scientific). Loaded peptides were concentrated on a trapping column (Acclaim C18 PepMap100, 100 μm, 2 cm) using 0.1% TFA at a flowrate of 10 μL·min^−1^. For sample separation a reversed phase column (Acclaim C18 PepMap100, 75 μm 50 cm) using a binary gradient was used (5% solvent B (84% ACN with 0.1% TFA) for 3 min, a linear increase in solvent B to 25% for 102 min, a linear increase in solvent B to 33% for 10 min, a linear increase in solvent B to 95% for 2 min followed by a linear decrease in solvent B to 5% for 5 min). MS survey scans were acquired using the following settings: mass spectrometer was operated in data‐dependent acquisition mode (DDA) with full MS scans from 300 to 1600 *m*/*z* at a resolution of 60 000 using the polysiloxane ion at 371.10124 *m*/*z* as lock mass27. The automatic gain control (AGC) was set to 1E6 and the maximum injection time to 120 ms. The 15 most intense ions above a threshold ion count of 5E3 were selected for fragmentation at a normalized collision energy (nCE) of 27% in each cycle of the acquisition analysis, following each survey scan. Dynamic exclusion was set to 15 s. Fragment ions were acquired at a resolution of 15 000 with an AGC of 5E4 and a maximum injection time of 50 ms.

All MS raw data were processed with proteome discoverer software 2.5.0.400 (Thermo Scientific, Schwer, Germany) and searched in target/decoy mode against a human Uniprot database [downloaded on November 2019, UniProt (www.uniprot.org)] using Sequest HT algorithm. The search parameters were precursor and fragment ion tolerances of 10 ppm and 0.02 Da for MS and MS/MS, respectively; trypsin set as enzyme with a max. of 2 missed cleavages; carbamidomethylation of Cysteine set as fixed modification and oxidation of methionine set as dynamic modification; using Percolator false discovery rate set to 0.01 for both peptide and protein identification. A label‐free quantification (LFQ) analysis was performed including replicates (no. stated in corresponding tables) for each condition. Proteins identified with ≥ 2 unique peptides and a log2 ratio ≥ 1 (XX‐fold enrichment), *P* ≥ 0.05 was considered as significantly regulated. This method has been described in [34].

### 2.17 Gene ontology

The data, derived from CoIP followed by LC–MS experiment, containing lists of proteins, were imported into the R workspace using the readxl package. The biomaRt package was employed to query Ensembl for gene symbols and Ensembl IDs of protein‐coding genes. Gene ontology enrichment analysis was carried out using the enrichGO() function from the clusterProfiler package, and results were filtered based on an adjusted *P*‐value (< 0.05) and a minimum count [3]. A list of keywords of interest was generated, containing the terms “chromatin”, “chromosome”, “nucleosome”, “DNA”, and “RNA”. The data were then filtered to include only the rows that contain these keywords in the “Description” column. A further filter was applied to exclude rows with the value “MF” in the “Ontology” column. Finally, a dotplot was created using ggplot2, highlighting term count as a function of adjusted *P*‐value (−log_2_).

## 3 Results

In our study, we conducted a series of experiments using a diverse panel of head and neck cancer (HNC) cell lines, A‐253, D‐562, FaDu, PCI 52, SCC‐9, and SCC‐15. These cell lines were selected to ensure the robustness and validity of our findings, even though we illustrate representative results in selected cell lines.

**Fig. 1:**
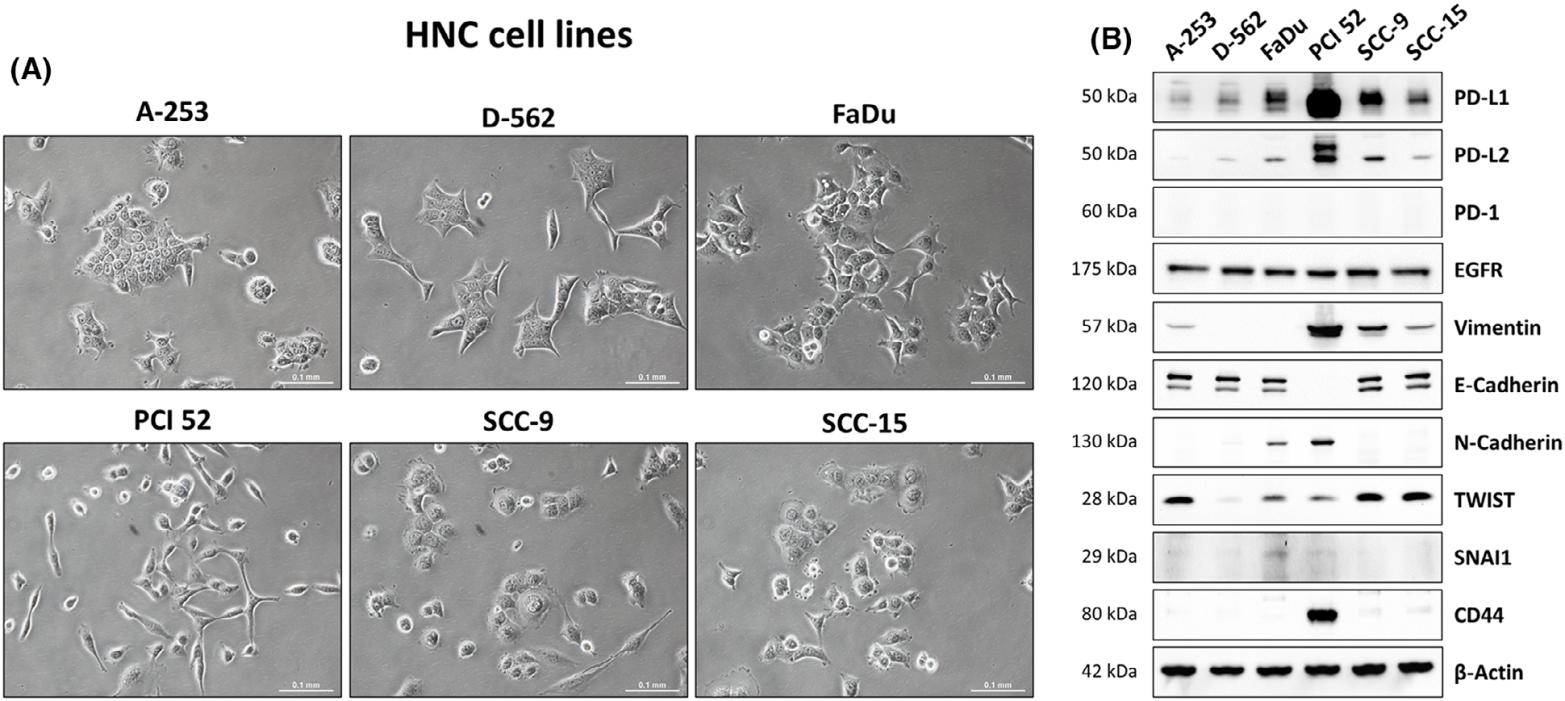
Characterization of HNC (head and neck cancer) cell lines used for this study. Analyzed cell lines: A‐253, D‐562, FaDu, PCI 52, SCC‐9, and SCC‐15. (A) Microscopic images of subconfluent, adherent cells. Magnification = 20x. Scale bar = 0.1 mm. *N* = 3. (B) WB (Western blot) analysis of tumor marker expression. For analysis, 30 μg of protein lysate was loaded onto a 10% acrylamide gel. Analyzed markers were PD‐L1, PD‐L2, PD‐1, EGFR, vimentin, E‐Cadherin, N‐Cadherin, TWIST, SNAI1 and CD44. β‐Actin served as loading control. *N* = 3. Further information about the cell lines is presented in Fig. S1.

Figure 1 provides a comprehensive overview of the cellular characteristics and tumor marker expression profiles of the used cell lines. We examined their unique cell morphology characteristics (A) and analyzed a wide range of tumor markers (B), including the PD‐1/PD‐L1 status, PD‐L2 expression, EMT markers (vimentin, E‐cadherin, and N‐cadherin), oncogenes (TWIST and Snai1), and the HNC stem cell marker CD44.

Our analysis revealed a wide spectrum of cellular characteristics and differentiation statuses among the analyzed HNC cell lines. These cell lines exhibited a continuum from more differentiated, epithelial characteristics with lower PD‐L1 expression (A‐253, D‐562, SCC‐15) to less differentiated, mesenchymal characteristics with higher PD‐L1 expression (FaDu, PCI 52, SCC‐9). Notably, marker expression closely correlated with cell morphology: A‐253, D‐562, and SCC‐15 displayed a typical cuboidal epithelial morphology, while FaDu, PCI 52, and SCC‐9 exhibited a rather spindle‐shaped mesenchymal cell morphology.

The analyzed cell lines show substantial heterogeneity in cellular characteristics. This diversity underscores the significance of our study and its potential implications for understanding HNC cell functions and therapeutic strategies.

For a detailed analysis of PD‐L1 expression in HNC cells, we conducted subcellular protein fractionation. This method provides valuable insights into protein expression across different cellular compartments, even in cases of low abundance. We performed this analysis in a total of six HNC cell lines and seven primary tumors of HNSCC patients.

**Fig. 2:**
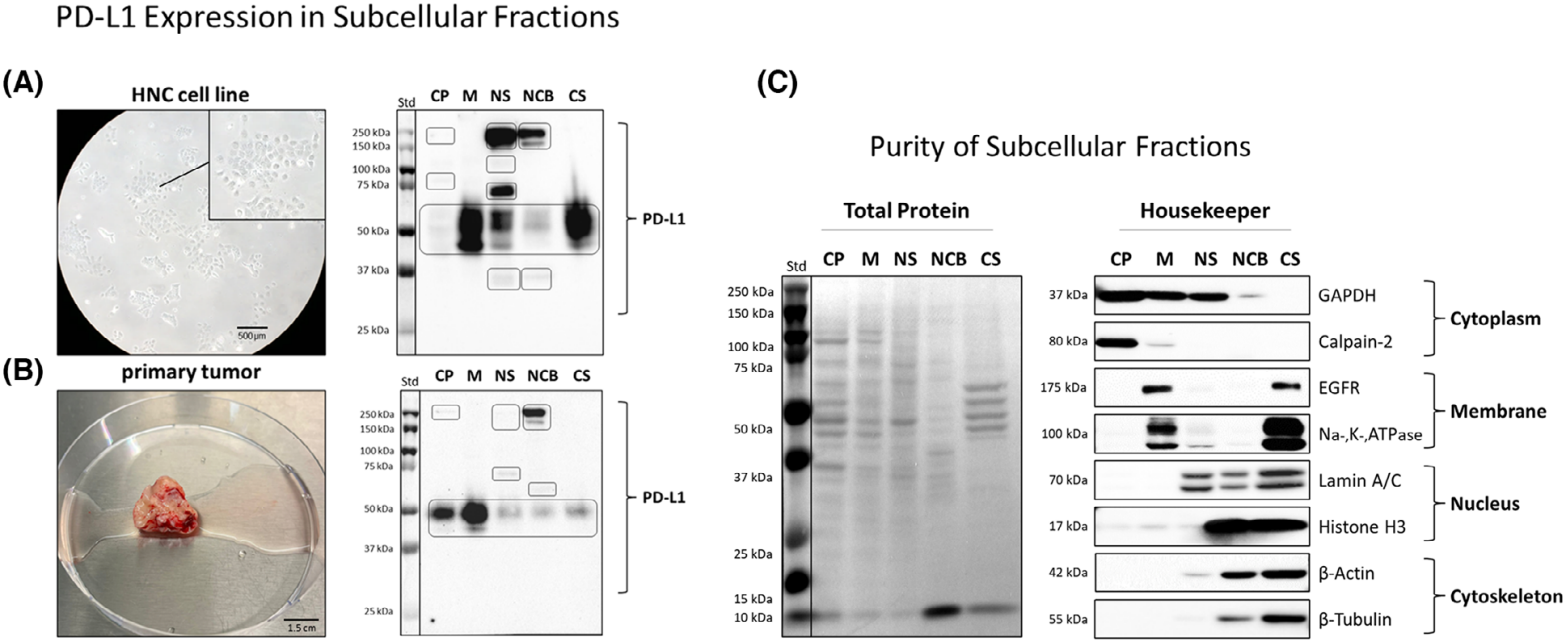
Immunodetection of PD‐L1 in subcellular protein fractions of HNC cells. Cells are separated into cytoplasmic (CP), membrane (M), nuclear soluble (NS), nuclear chromatin‐bound (NCB) and cytoskeletal (CS) protein fraction. (A) PD‐L1 detection *in vitro*, with cell line SCC‐9 as representative. Scale bar 500 μm. (B) PD‐L1 detection in a primary tumor from an HNSCC (head and neck squamous cell carcinoma) patient (PT#1) as representative. Scale bar 1.5 cm. (C) Purity validation of subcellular protein fractions via marker expression of GAPDH, calpain‐2, EGFR, Na‐, K‐, ATPase, lamin A/C, histone H3, β‐actin, and β‐tubulin. Ponceau S was performed for total protein staining. For WB (Western blot) analysis, 20 μg of protein was loaded onto a 10% acrylamide gel. Immunodetection of PD‐L1 expression in subcellular fractions of HNC (head and neck cancer) cell line A‐253, D‐562, FaDu, PCI 52, SCC‐9 and SCC‐15 are shown in Fig. S1 including original blots, as well as HNSCC primary tumors PT#1 – PT#8. Figure S1 also provides further information about origin, differentiation status, and PD‐L1 expression patterns of HNC cell lines and primary tumors. Analyzed HNC cell lines *n* = 6. Analyzed HNSCC primary tumors *n* = 7. For original blots of PCI 52 subcellular fractionation purity validation and purity validation for FaDu and SCC‐9, refer to Fig. S2.

Figure 2 presents immunodetection of PD‐L1 in protein fractions obtained from the cytoplasm (CP), membrane (M), nuclear soluble (NS), nuclear chromatin‐bound (NCB), and cytoskeleton (CS) of the representative HNC cell line SCC‐9 (A), as well as a representative primary tumor sample of a HNSCC patient (B). PD‐L1 was not only detectable in the membrane protein fraction but in all analyzed subcellular fractions, including the nuclear fractions. Different expression levels of PD‐L1 were observed depending on the cellular fraction analyzed, despite equal protein loading. Similar banding patterns were observed between *in vitro* and primary tumor samples. Both samples exhibited the presence of standard forms of PD‐L1, well known as unglycosylated and glycosylated state of PD‐L1, ranging from 40 to 55 kDa. Additionally, novel PD‐L1 variants with higher molecular weight bands of ~ 70 kDa were detected in the NS fraction. In both the NS and NCB fractions, PD‐L1 bands with higher molecular weight, ranging from approximately 150 to 180 kDa, were observed. The origin of the high molecular weight band is still unknown. Several potential factors could contribute to this phenomenon, including post‐translational modifications, oligomerization, or interaction with binding partners. Further analysis in this manuscript leads to the assumption that it is due to protein multimerization. Subcellular protein fractionation and immunodetection of all six analyzed HNC cell lines and seven primary tumor samples from HNSCC patients are shown in Fig. S1, including additional information about their origin and cellular tumor characteristics. In these samples, similar PD‐L1 expression patterns were observed.

To validate subcellular protein fraction purity, we examined marker protein expression associated with different cellular compartments (C). GAPDH and Calpain‐2 were used as cytoplasmic markers, EGFR and Na‐, K‐, ATPase as membrane markers, lamim A/C and histone H3 as nuclear markers, and β‐actin and β‐tubulin as cytoskeletal markers. Total protein staining using Ponceau S was performed to visualize and assess the total protein content. Marker expression significantly varied depending on the analyzed protein fraction, confirming distinct subcellular localizations and indicating the purity of the cellular protein fractions. Minimal cross‐contamination further supports the reliability of the subcellular fractionation method, ensuring accurate subcellular protein localization characterization. Original blots of the validation of subcellular protein fraction purity of PCI 52 is shown in Fig. S2, including the validation of subcellular protein fraction purity of the cell lines FaDu and SCC‐9.

In addition to the ‘Subcellular Protein Fractionation Kits’ from Thermo Scientific, we also employed an alternative subcellular fractionation method, using the ‘Subcellular Fractionation Protocol’ from Abcam, to prove reliability of our findings. The results are shown in Fig. S3. This approach enabled the separation of cells into cytoplasmic, membrane and nuclear protein fractions. This alternative method independently confirmed the presence of nuclear PD‐L1 variants, validating the findings presented in Fig. 2.

**Fig. 3:**
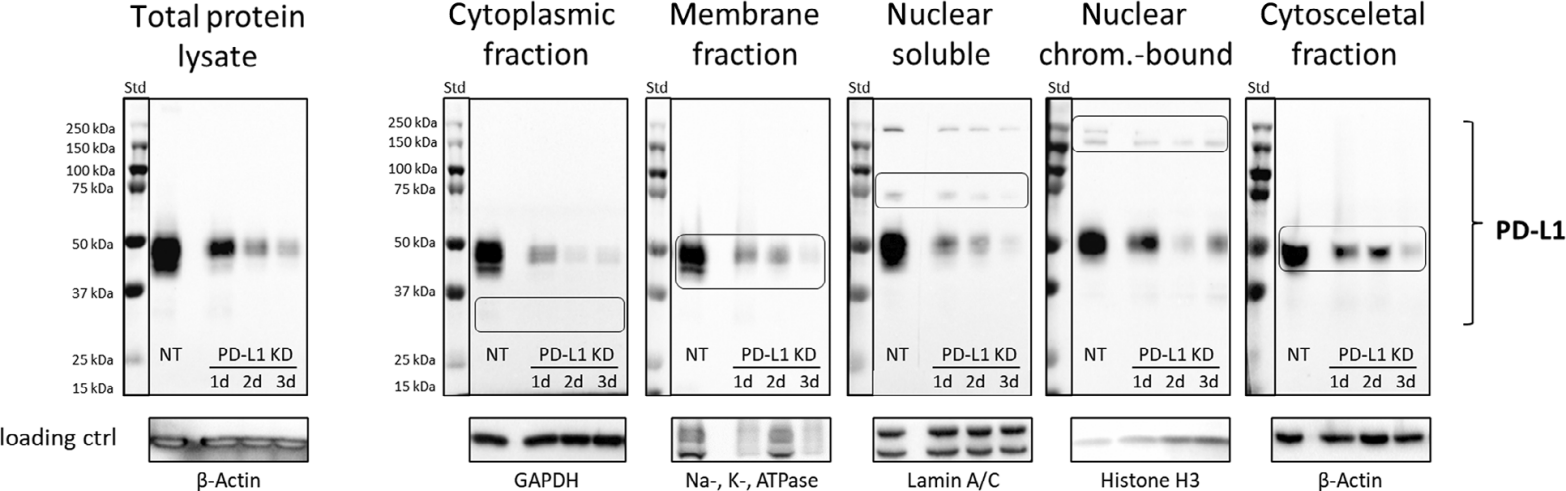
Specificity of PD‐L1 immunodetection. Time course of decreasing PD‐L1 immunodetection after transient PD‐L1 siRNA knockdown (KD) in different cellular fractions of representative HNC (head and neck cancer) cell line PCI 52. Non‐targeting siRNA (NT) after 3 days (d) incubation was used as transfection control. Total cell lysates and subcellular protein fractions are shown on separate blots including the respective housekeeping protein β‐Actin, GAPDH, Na‐, K‐, ATPase, Lamin A/C, or Histone H3 as loading controls. For WB analysis of siRNA KD, 20 μg of protein was loaded onto a 10% acrylamide gel. Tested cell lines: Fadu, PCI 52, *n* = 2. For original blots, see Fig. S4.

Figure 3 demonstrates the specificity of PD‐L1 immunodetection using the monoclonal anti‐PD‐L1 (E1L3N) XP^®^ rabbit antibody from Cell Signaling Technologies, which recognizes endogenous levels of total human PD‐L1 protein near the carboxy‐terminus. A transient PD‐L1 siRNA knockdown (KD) experiment shows the time course of PD‐L1 expression after 1, 2, and 3 days after siRNA transfection, followed by immunodetection. Transfection with non‐targeting siRNA (NT) was used as a control. The distinct cellular fractions of the representative HNC cell line PCI 52 are shown with respective loading controls. Total protein cell lysates, CP, M, and CS protein fractions exhibit decreasing PD‐L1 expression at 40 to 55 kDa within 3 days of PD‐L1 KD compared to the NT control after 3 days. The NS protein fraction reveals decreasing PD‐L1 immunodetection at sizes of ~70 kDa and 180 kDa. The NCB protein fraction displays a double banding at about 150 and 180 kDa, where the 180 kDa band decreases within 3 days of siRNA KD. The 150 kDa band remains more stable over the observed time course. Figure S4 presents the original blot after transient PD‐L1 siRNA KD of HNC cell lines PCI 52 and FaDu, over a time period of 8 days. Every 24 h (h) cells were harvested for PD‐L1 protein expression analysis.

**Fig. 4:**
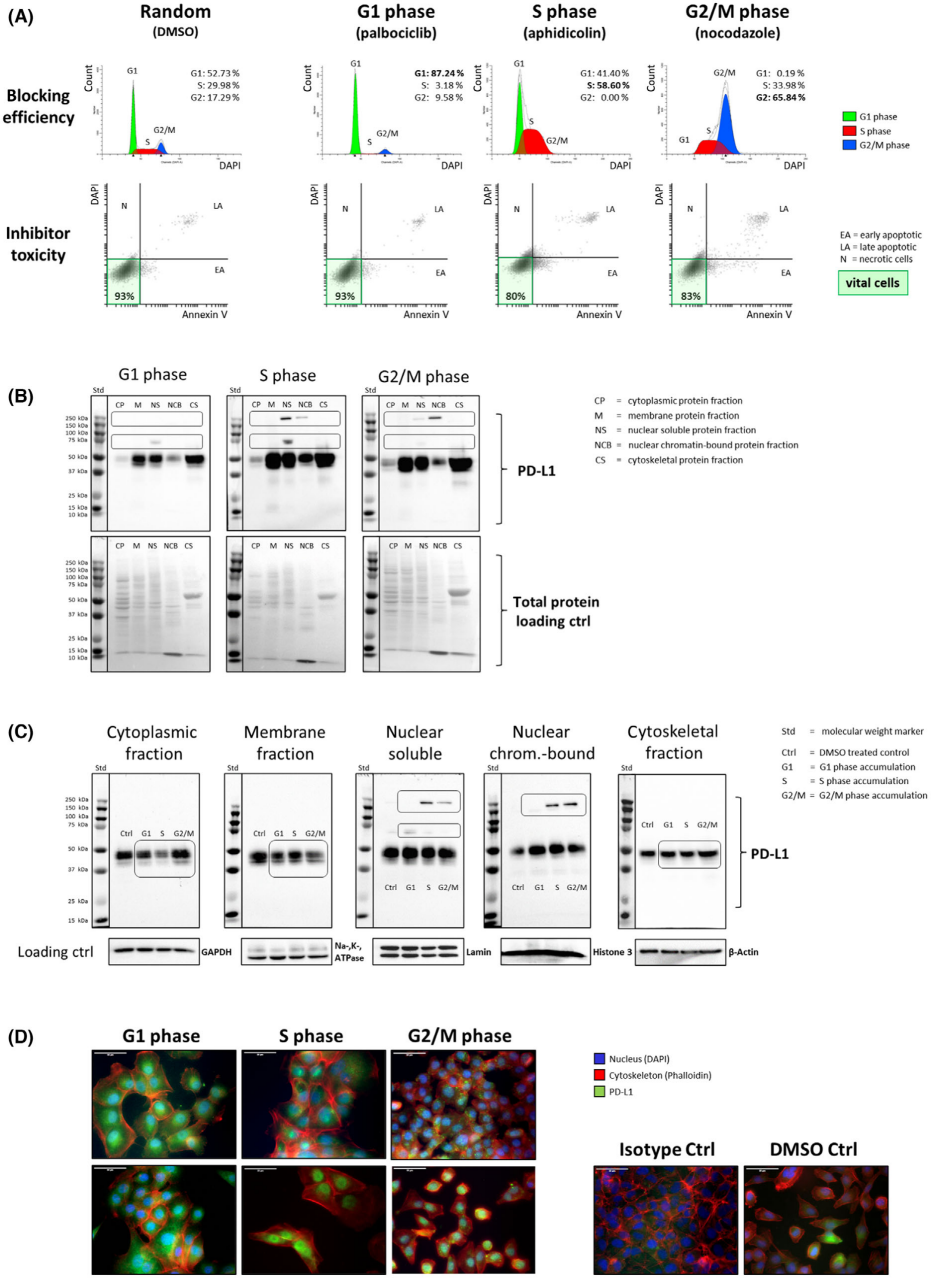
Cell cycle dependent PD‐L1 expression and cellular localization. (A) Flow cytometric analysis of HNC (head and neck cancer) cell line FaDu, as representative, after cell cycle inhibition. Cell cycle progression was blocked at the G1, S or G2/M phase with 50 nm palbociclib for 12 h, 1000 nm aphidicolin for 24 h or 50 nm nocodazole for 12 h, respectively. As a control, cells were treated with 0.03% DMSO for 24 h. The efficiency of cell cycle blockade was demonstrated by S phase analysis using DAPI to quantify the DNA content in each cell. Inhibitor toxicity was determined using DAPI/Annexin V double staining to identify the proportion of vital cells in the cell population in percentage [%], illustrated as dot plots. Per sample, 5 × 10^4^ cells were analyzed. Tested cell lines (*n* = 6). To achieve optimal conditions, these experiments were performed separately for each cell line studied. (B) Exemplary WB (Western blot) analysis of PD‐L1 banding patterns in cellular fractions in HNC cell line PCI 52 blocked with 125 nm palbociclib for 12 h in G1 phase, with 1200 nm aphidicolin for 24 h in S phase and with 480 nm nocodazole for 12 h in G2/M phase. Total protein expression with Ponceau S staining solution was used as loading control. 20 μg of total protein was loaded. For original blots including analysis of HNC cell line FaDu, see Fig. S5. Tested cell lines *n* = 2. (C) Signal intensity of PD‐L1 expression in the respective cellular fraction of the representative HNC cell line PCI 52 during cell cycle progression. For original blots, including analysis of HNC cell line FaDu, see Fig. S5. Tested cell lines *n* = 2. (D) Immunofluorescence staining of PD‐L1 in the HNC cell line PCI 52, blocked in G1, S, and G2/M phase. Magnification = 40x, scale bar = 50 μm. Assay performed in duplicates. On each slide, *n* = 10 pictures were taken for documentation. Figure S5 provides the displayed images with a highlighted nuclear border.

To investigate whether the expression of PD‐L1 variants is dependent on the cell cycle, cell cycle progression was arrested at different phases using specific inhibitors, as shown in Fig. 4. In this study, cells of the HNC cell line FaDu were treated with inhibitors targeting G1, S, or G2/M phase. Cells treated with the solvent DMSO served as controls.

A presents the cell cycle blocking efficiency via flow cytometric S phase analysis (upper row of histograms) and the potential inhibitor toxicity (lower row of dot plots). Treatment with DMSO did not significantly affect cell cycle progression, resulting in a random distribution of cells across all phases of the cell cycle. Analysis of blocking efficiency demonstrated specific inhibition of cell cycle progression, resulting in the accumulation of cells in G1 (green), S (red), or G2/M phase (blue) in response to the respective inhibitors. The efficacy of cell cycle inhibition varied depending on the concentration of the inhibitor used, with higher concentrations showing increased toxicity. Balance was achieved by selecting inhibitor concentrations that effectively arrested cell cycle progression while maintaining low toxicity. Effective cell cycle blockade was characterized by a significant accumulation of cells in the corresponding cell cycle phase. Cell viability, highlighted in the green quadrant, was at least 80% following each cell cycle inhibition, while control cell viability was 93%.

Furthermore, we aimed to investigate the specific expression patterns of PD‐L1 variants during distinct cell cycle stages. We arrested cell cycle progression of HNC cell lines at G1, S, or G2/M phase and performed cell fractionation followed by protein expression analysis via WB. B displays three blots of the cell line PCI 52. Each blot represents the expression of PD‐L1 in subcellular fractions of cells blocked at G1, S, or G2/M phase of the cell cycle. These blots reveal distinct PD‐L1 banding patterns in subcellular fractions of HNC cell lines during cell cycle progression. The standard molecular weight between 40 and 55 kDa was observed in all cell cycle phases. PD‐L1 expression with a molecular weight exceeding 150 kDa was exclusively detectable in S and G2/M phase, while it was not detectable in the G1 phase. PD‐L1 with a molecular weight of ~ 70 kDa was detectable at all cell cycle stages. Figure S5 includes the original blots from the analysis of two HNC cell lines PCI 52 and FaDu.

In order to detect changes in signal intensity within specific cellular fractions during cell cycle progression, cell lysates from cells at different cell cycle phases were applied to the same blot (**C**). Cells treated with DMSO were used as control (Ctrl). Treatment with DMSO did not have a significant effect on cell cycle progression, resulting in random distribution of cells across all cell cycle phases. The PD‐L1 variant with a molecular weight of 40–55 kDa exhibited its highest expression on the cell membrane during the S phase, compared to the G1 and G2/M phases, while its expression in the cytoplasmic protein fraction was reduced during the S phase. Additionally, this figure demonstrates that the PD‐L1 variants with ~ 70 kDa as well as > 150 kDa are exclusively expressed in nuclear fractions. PD‐L1 with a molecular weight of ~ 70 kDa is only expressed in the NS protein fraction. 70 kDa PD‐L1 shows the highest expression in G1 phase, which is reduced during cell cycle progression. The PD‐L1 variant > 150 kDa is expressed in both the NS and NCB protein fractions. It is absent during the G1 phase, appears in the NS fraction during the S phase, and accumulates as NCB PD‐L1 as the cell cycle progresses to the G2/M phase. The expression of the 40–55 kDa PD‐L1 variant is reduced in the nuclear fractions during the G2/M phase. However, the expression of PD‐L1 with a molecular weight > 150 kDa increases the overall PD‐L1 expression in the nucleus during the S and G2/M phases. Figure S5 includes the original blots from the analysis of two HNC cell lines PCI 52 and FaDu.

In D, we present immunofluorescence staining of PD‐L1 in synchronized HNC cells at different cell cycle phases. As controls, we include cells treated with DMSO, resulting in a random distribution of cell cycle phases, as well as cells incubated with a respective IgG isotype control antibody to assess unspecific antibody signals. Notably, cell cycle inhibition leads to distinct cellular morphology as cells progress through the cell cycle. Specifically, cells enlarge during the S phase and become smaller during the G2/M phase. In the G1 phase, PD‐L1 exhibits a diffuse distribution throughout the cells. As the cell cycle progresses, a distinct translocation of PD‐L1 is observed, with its localization shifting towards and within the nuclei. Notably, during the S phase, PD‐L1 distribution near/in the nuclei adopts a more confined pattern compared to G1 phase, forming torus or condensed chromatin‐like structures. Meanwhile, in the G2/M phase, PD‐L1 distribution is characterized by the presence of distinct ‘hotspots.’ Figure S5 showcases the presented images with a highlighted nuclear border, as well as images focusing solely on PD‐L1 detection for an optimal representation of dynamic PD‐L1 localization during cell cycle progression.

**Fig. 5:**
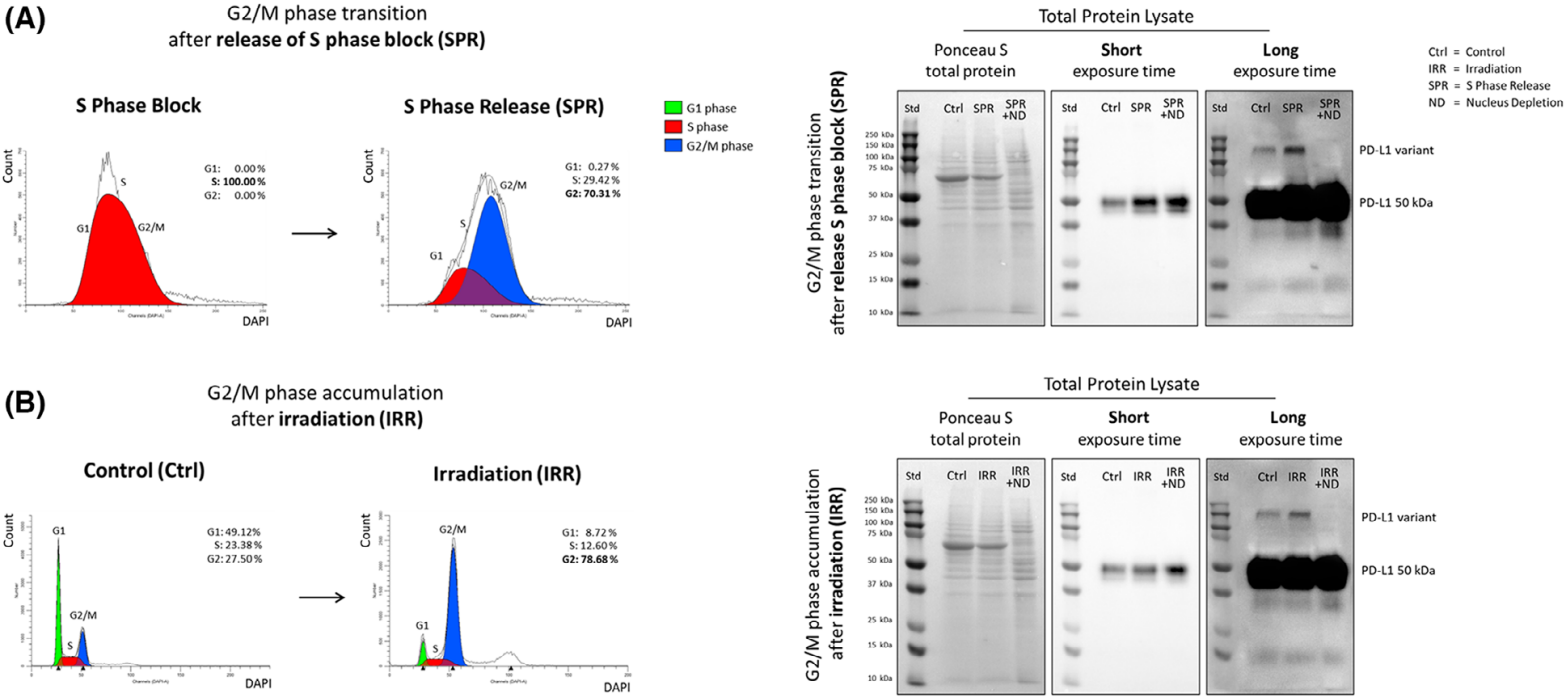
PD‐L1 variant detection in total protein lysate. Experiments were performed with HNC (head and neck cancer) cell line PCI 52. (A) Increase of PD‐L1 variant detection after G2 phase transition due to S phase release (SPR) or (B) G2 phase accumulation after irradiation (IRR). Analysis 16 h after irradiation with 8 Gy. A non‐treated cells served as control (Ctrl). For performance of S phase release experiments, cells were first blocked with aphidicolin. Inhibitor removal was done with a replacement of cell culture medium. Analysis was performed 3 h after inhibitor removal. Nuclei were depleted after Fritsch et al. [32] (ND). Flow cytometric analysis was used for identification of cell cycle phase distribution. *N* = 3. For WB (Western blot) analysis 20 μg protein was loaded on a 10% acrylamide gel. Ponceau S staining serves as loading control. Stronger signal detection of PD‐L1 variants in total protein lysate was achieved by longer exposure time and signal enhancement. *N* = 2.

Detection of high molecular weight PD‐L1 bands in total protein lysates is challenging due to dynamic appearance, low abundance, and limited sensitivity of Western blot analysis. Figure 5 demonstrates that an extended exposure time and brightness adjustment enhance the detection of novel PD‐L1 variants in total protein lysate.

The aim of this experiment was to induce the expression of high molecular weight nuclear PD‐L1 by accumulating cells in the G2/M phase. (A) Here, instead of the cell cycle inhibitor nocodazole, which blocks cells in G2/M, we used aphidicolin and took advantage of the reversibility of its inhibitory effect. Three hours after inhibitor removal (S phase release, SPR), the majority of cells transitioned from S to G2 phase (70.31%). The accumulation of cells in G2 phase resulted in an increase in PD‐L1 total protein expression, as well as an increase in signal intensity of the high molecular weight PD‐L1 band.

Irrespective of inhibitor treatment, we utilized irradiation as an alternative method to accumulate cells in the G2 phase of the cell cycle (B). We irradiated cells with 8 Gy (IRR) and analyzed them 16 h after irradiation treatment. For control, non‐irradiated cells were used (Ctrl). 16 h after irradiation cells accumulated in G2 phase (78.68%) due to DNA damage. The accumulation of cells in G2 phase not only resulted in an increase in PD‐L1 total protein expression but also in an increase of signal intensity of the high molecular weight PD‐L1 band.

In summary, both approaches induced accumulation of cells in G2 phase and increased the total PD‐L1 expression, including the high molecular weight PD‐L1 variant, compared to the non‐treated control. Furthermore, the signal reduction of the high molecular weight PD‐L1 bands following the depletion of nuclei (ND) substantiates the nuclear localization of this variant.

**Fig. 6:**
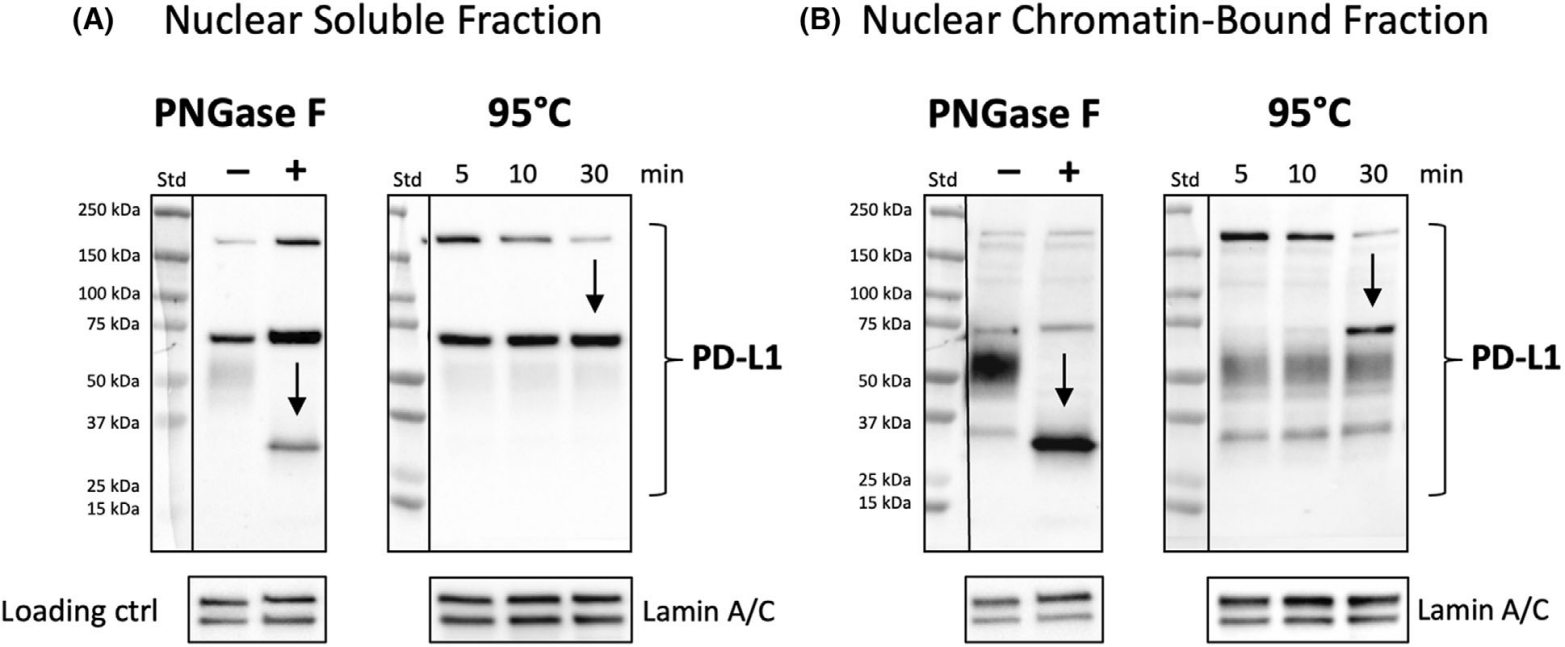
Origin of PD‐L1 variants with high molecular weight. Deglycosylation via PNGase F digestion (A) and excessive heating for disruption of covalent bonds between possible interacting partners (B) was performed in NS (nuclear soluble) and NCB (nuclear chromatin bound) protein fraction samples of HNC cell line FaDu. *N* = 4. For each treatment, 20 μg protein was used and loaded onto a 4–12% gradient gel. Lamin A/C was used as loading control. For original blots and further investigations (Endo H digestion, additional heating duration and temperature), see Fig. S6.

So far, we have reported the identification of unusual high‐molecular‐weight nuclear PD‐L1 variants in HNC cells and confirmed their specificity. In Fig. 6, our objective was to explore the underlying cause for the high molecular weight of these newly detected PD‐L1 variants. Specifically, we sought to determine whether the molecular weight differences observed in the NS and NCB protein fractions, with bands at approx. 70 kDa and over 150 kDa, were due to extensive glycosylation or PD‐L1 multimerization.

To investigate this, we employed PNGase F to cleave N‐linked oligosaccharides. Interestingly, in the NS protein fraction, immunodetection of both the > 150 kDa and ~ 70 kDa PD‐L1 bands became more pronounced after digestion (+) compared to the control (−). This could be attributed to enhanced antibody binding site accessibility following deglycosylation [35]. Conversely, in the NCB protein fraction, immunodetection of PD‐L1 bands > 150 and ~ 70 kDa remained relatively consistent after N‐linked oligosaccharide digestion. Therefore, the high molecular weight of PD‐L1 due to extensive glycosylation appears less likely. An observable shift in molecular weight occurred, with bands moving from 40 to 55 kDa to approximately 34 kDa in both the NS and NCB protein fractions. For original blots and additional Endo H digestion analysis of *N*‐acetylglucosamine, refer to Fig. S6.

As an alternative approach, the NS and NCB protein fractions were subjected to heating at 95 °C for 5, 10, and 30 min, aimed at disrupting covalent bonds between potential interacting partners. As a result of this treatment, immunodetection of PD‐L1 bands > 150 kDa decreased over time, while immunodetection of the ~ 70 kDa band intensified. Immunodetection of PD‐L1 bands ranging from 40 to 55 kDa remained relatively stable during heating at 95 °C for 5–30 min. This specific pattern observed following heat disruption suggests that PD‐L1 multimerization is the likely cause of the high molecular weight. For original blots and details regarding heating procedures for 5, 10, 30, and 60 min at 70 and 95 °C, refer to Fig. S6.

**Fig. 7:**
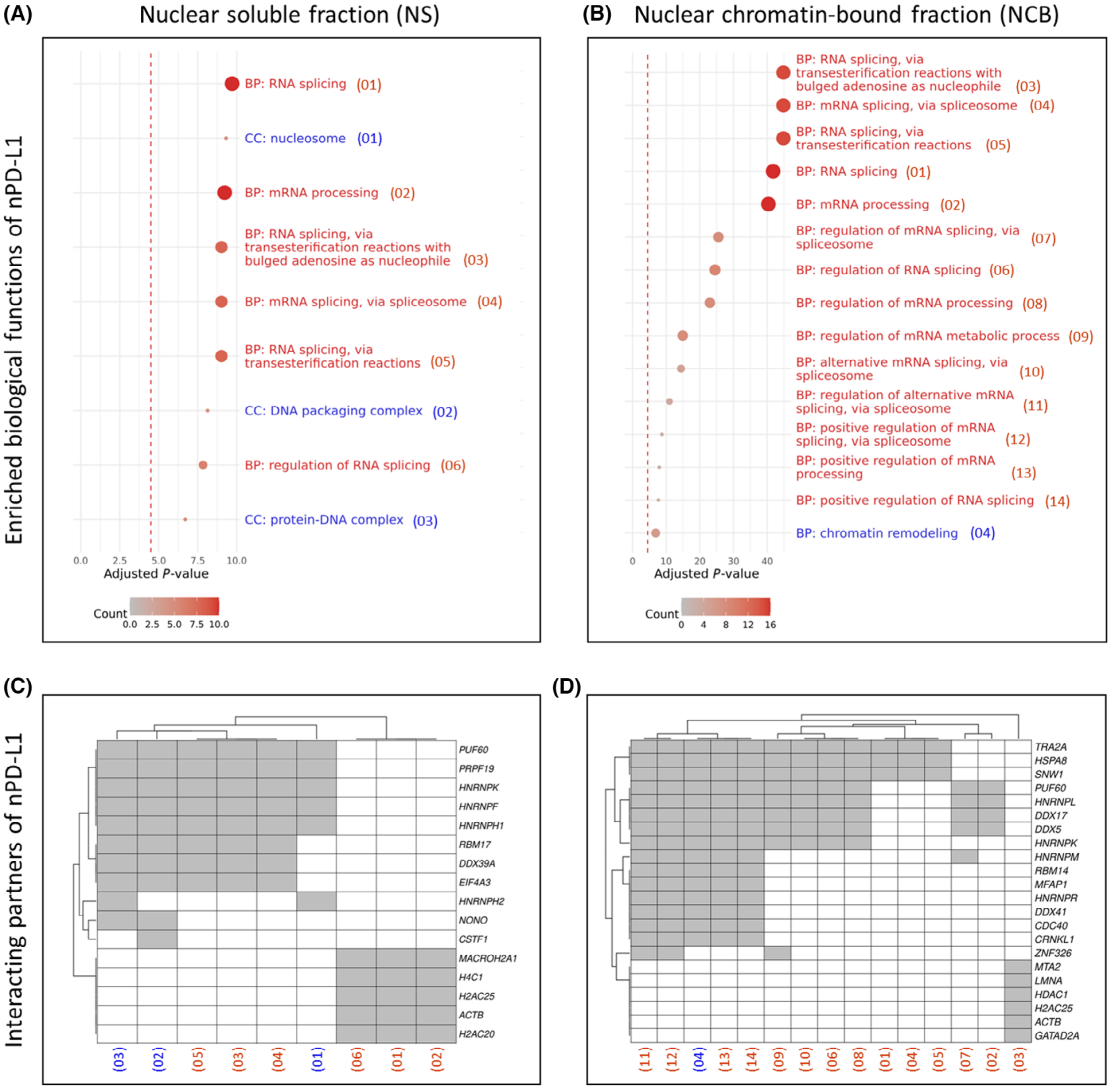
Involvement of nuclear PD‐L1 in biological processes like DNA remodeling and mRNA splicing. Gene ontology analysis reveals involvement of nuclear PD‐L1 in biological processes (BP) and cellular components (CC) associated with DNA remodeling (indicated in blue) and mRNA splicing (indicated in red) in the HNC cell lines FaDu (A) and PCI 52 (B). The size and color of spheres represent the total number of genes whose encoded proteins interact with PD‐L1 and are part of the ontology term. The dashed vertical red line marks the threshold of statistical significance (adjusted *P* < 0.05). Additionally, we present a list of individual PD‐L1 interacting partners in HNC (head and neck cancer) cell lines FaDu (C) and PCI 52 (D), correlated with their roles in biological processes and cellular components. A total of 100 μg of total protein per fraction was employed for the CoIP procedure, with a specific PD‐L1 antibody used for PD‐L1 capturing. Proteins captured with a respective IgG isotype control antibody were considered unspecific and excluded from the analysis. For original blots following CoIP and a list of individually detected proteins, refer to Fig. S7.

To investigate the nuclear functions of PD‐L1, we conducted a CoIP experiment using the nuclear fractions of HNC cell lines FaDu and PCI 52, aiming to identify interacting partners. PD‐L1 was selectively captured with a specific PD‐L1 antibody, and the eluate was subsequently subjected to LC–MS analysis. The results of this analysis were further explored through a gene ontology analysis, presented in Fig. 7.

Within the nuclear fractions, we observed PD‐L1 interactions with proteins primarily associated with DNA remodeling and mRNA splicing. In the nuclear soluble protein fraction, the proteins were related to the biological process (BP) of RNA splicing and processing, alongside cellular components such as the nucleosome, the DNA packaging complex, and protein‐DNA complexes (A). In the nuclear chromatin‐bound protein fraction, we identified proteins primarily related to RNA processing, as well as proteins associated with chromatin remodeling (B). Detailed lists of PD‐L1 interactions with soluble proteins (C) and chromatin‐bound proteins (D) in the nucleus are provided below. For original blots of PD‐L1 detection after CoIP and a list of PD‐L1 interacting partners found in mass spectrometry analysis, refer to Fig. S7.

**Fig. 8:**
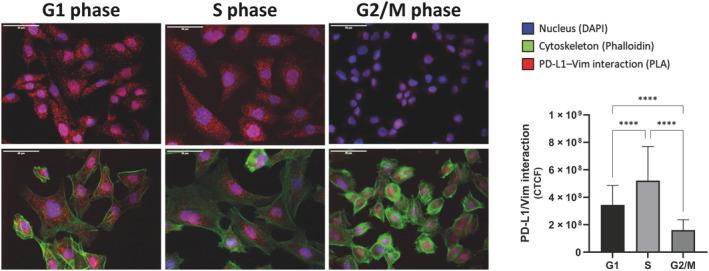
Cell cycle‐dependent interaction of PD‐L1 with Vimentin. Proximity ligation assay (PLA) in HNC (head and neck cancer) cell line PCI 52 was performed to visualize the interaction of PD‐L1 with Vimentin as red dots. Cells are synchronized in G1, S or G2/M phase. Nuclei are stained with DAPI (blue), cytoskeleton with Phalloidin (green). In order to focus on the PD‐L1/Vimentin interaction, images in the upper row are displayed without the cytoskeleton staining. For determination of PD‐L1 localization, triple staining was performed in the lower row. A respective IgG isotype antibody was used as control is shown in Fig. S8. Figure S8 presents the displayed images with a highlighted nuclear border and the images used for quantification. Magnification = 40×, scale bar = 50 μm. For quantification, the fluorescence intensity of PLA (proximity ligation) signal (red) was measured by Fiji ImageJ software. The corrected total intensity was calculated by subtracting the mean background in relation to the area analyzed (CTCF). The results are expressed as means ± SD (standard deviation). G1: *n* = 47, S: *n* = 32, G2/M: *n* = 58. One‐way ANOVA with multiple comparisons (****P < 0.0001).

LC–MS analysis revealed specific interactions of PD‐L1 with cytoskeletal proteins (Fig. S7). This finding aligns with the recently published data by Gao et al., who highlighted alternative mechanisms by which membrane proteins can translocate to the nucleus, independent of traditional nuclear localization signals. Notably, vimentin and Importin α1 have been identified as shuttles involved in the nucleo‐cytoplasmac shuttling of PD‐L1 into the nucleus [27]. Consequently, we aimed to investigate the potential interaction between PD‐L1 and vimentin in HNC cells and determine if this interaction varies across different cell cycle phases, shown in Fig. 8.

Using proximity ligation assays (PLAs), we observed strong interaction between PD‐L1 and vimentin, indicated as red dots. Cell cycle inhibition revealed a dynamic interaction of PD‐L1 and vimentin during cell cycle progression. The interaction increased after transition from G1 to S phase and decreased during G2/M phase. Furthermore, immunofluorescent pictures show a locational change of PD‐L1 and vimentin interaction during cell cycle progression. In G1 phase, the interaction is distributed rather equally within the cell, while in S phase the interaction is centered to the nucleus. These findings provide novel insights into the specific cell cycle‐dependent interactions of PD‐L1, likely facilitating its cytoplasmic shuttling. Figure S8 includes additional images, with the presented ones highlighted by a nuclear border. Images exclusively displaying the PLA signal are provided for an enhanced presentation of interaction localization.

## 4 Discussion

The interaction between PD‐L1 and its receptor PD‐1 has been extensively studied as a ligand‐receptor pair. In addition to its immunoregulatory function, emerging evidence suggests that PD‐L1 also exerts cell‐intrinsic effects on cancer cell signaling. Similarly, the PD‐1 receptor has been found to have cell‐intrinsic effects. For instance, it regulates the mechanistic target of rapamycin (mTOR) signaling pathway and promotes tumor growth in melanoma cells [36]. Recent investigations have focused on the localization of PD‐L1 in various cellular compartments, including subcellular fractions and the extracellular space. These include nuclear PD‐L1 (nPD‐L1), cytoplasmic PD‐L1 (cPD‐L1), soluble PD‐L1 (sPD‐L1), and PD‐L1 present in extracellular vesicles (EV PD‐L1). Understanding the role of these non‐cytomembrane PD‐L1 variants is crucial for unraveling the mechanisms of underlying resistance to anti‐PD‐1/PD‐L1 therapy. Importantly, these PD‐L1 variants hold significant diagnostic value in cancer and represent promising therapeutic targets for cancer treatment [37].

Subcellular fractionation is a valuable technique that not only allows the detection of proteins at various cellular locations but also the detection of proteins at low concentrations that may otherwise remain undetectable in the total protein lysate when analyzed by Western blotting. Using this technique, we were able to detect PD‐L1 not only in the membrane protein fraction but also in all analyzed subcellular fractions, including the nuclear fractions. Across the six HNC cell lines and in seven primary HNSCC tissue samples, we observed consistent expression patterns of PD‐L1 (Fig. S1). The presence of PD‐L1 variants at specific molecular weights suggests the existence of potential regulatory mechanisms and highlights the importance of subcellular localization in PD‐L1 expression in HNC. Notably, PD‐L1 was not limited to its standard forms as unglycosylated and glycosylated forms ranging from 40 to 55 kDa [38]. Additionally, higher molecular weight bands were observed in the nuclear fractions (~ 70 kDa and 150–180 kDa). Moreover, our data are supported by Yu et al. who found that nuclear PD‐L1 was super‐shifted (over 150 kDa) compared to the cytosolic/membrane fraction by Western blot, and this band was absent in PD‐L1 knockout cells. Furthermore, they describe the same ‘super‐shifted’ bands for nuclear PD‐L1 in multiple cancer cells of different origins [28].

We further investigated the origin of high molecular weight PD‐L1 observed in the nuclear soluble and nuclear chromatin‐bound protein fractions, aiming to determine if it is attributed to extensive glycosylation or potential PD‐L1 multimerization. Post‐transcriptional modifications (PTMs) play a crucial role in determining protein localization and stability. PD‐L1 expression is regulated at multiple levels, including transcriptional regulation and PTMs, such as phosphorylation, ubiquitination, methylation, glycosylation, and palmitoylation [39]. For this purpose, cleavage of *N*‐linked oligosaccharides by PNGase F or Endo H, or excessive heating with different reducing reagents was performed. We were able to rule out the possibility that the heavy variant might be complex glycan forms by treatment with PNGase F and Endo H, as no change in band height occurred after treatment. However, after more intensive heat treatment, there was clearly a shift from the > 150 kDa variant to the ~ 70 kDa variant, which suggests that PD‐L1 may be involved in a multiprotein complex. The use of different reducing reagents, β‐mercaptoethanol (2‐ME) or diethiothreitol (DTT), alone or in combination, resulted in no difference of the PD‐L1 detection pattern (not shown). High molecular weight PD‐L1 is likely a result of PD‐L1 dimerization or multimerization with itself [40], or the formation of a complex between PD‐L1 and specific binding partners also described elsewhere [27, 28]. Although Yu et al. [28] discovered the 150 kDa band in various tumors, it has not yet been described in HNSCC until now. Moreover, we found a clear difference in expression between chromatin‐bound and nuclear soluble protein fraction. In addition, this difference was very evident after blockade of cells in individual cell cycle phases. In S phase, the ~ 70 kDa and the > 150 kDa variants were highly expressed in the nuclear soluble protein fraction. However, in G2/M phase, the ~ 70 kDa variant in the nuclear soluble protein fraction had disappeared, and the > 150 kDa variant was highly expressed in the nuclear chromatin‐bound protein fraction. As we have shown here, there is increasing evidence in the literature that PD‐L1 also functions as a nuclear protein [41, 42].

The nuclear localization of membrane proteins is not a novel phenomenon. For example, the presence of epidermal growth factor receptor (EGFR) family members in the nucleus has been very well documented [43, 44]. Unlike PD‐L1, EGFR has a nuclear localization signal (NLS), a short sequence that is recognized by importin proteins responsible for directing tagged proteins through the nuclear pore complex and into the nucleus [45]. However, no NLS has been identified for PD‐L1 so far. Consequently, alternative pathways may be involved in its nuclear translocation. Post‐translational modifications, such as glycosylation, could potentially play a role in this process [46]. Additionally, the precise mechanisms by which G protein‐coupled receptors localize to the nucleus remain unclear. Proposed mechanisms include small GTPase‐driven processes and involvement of sorting nexin proteins within the vesicular transport pathway [47]. Further investigation is needed to elucidate the precise mechanisms underlying the nuclear localization of PD‐L1. Our findings are supported by Gao et al., who propose that the nuclear translocation of PD‐L1 is regulated by a deacetylation mechanism involving p300‐mediated acetylation at K263 and HDAC2‐mediated deacetylation on the cytoplasmic side. They demonstrate that deletion of the C‐tail prevents PD‐L1 nuclear translocation, and interfering with PD‐L1 acetylation through genetic or pharmacological means inhibits its nuclear translocation. Additionally, vimentin has been described as a shuttle for PD‐L1 nuclear transport [22]. It has a positive relationship with PD‐L1 expression and promotes the epithelial‐mesenchymal transition (EMT) [48]. Moreover, positive vimentin expression on the cell surface showed a higher nPD‐L1 expression in multiple cancer types, which indicates the regulatory function of vimentin in PD‐L1 nuclear location [49].Gao et al. [27] propose a model in which deacetylation of PD‐L1 by HDAC2 on plasma membrane enables PD‐L1 to interact with HIP1R and cargo proteins for endocytosis and with vimentin to traffic through the cytoskeleton, and finally translocate into the nucleus. These findings strongly support our observations of a cell cycle‐dependent interaction between PD‐L1 and vimentin. Notably, the interaction is prominent during G1 and S phases, but diminishes in G2/M phase. Interestingly, it appears that the interaction transitions from a membrane/cytoplasmic interaction in G1 phase towards the nucleus in S phase. This suggests that the interaction may cease after S phase, possibly indicating the completion of PD‐L1 shuttling and its arrival at the desired location.

After confirming the nuclear localization of PD‐L1, its roles within the nucleus have become an area of significant research. Yu et al. have shown that nuclear PD‐L1 regulates sister chromatids as a subunit of the cohesin complex. A deficiency in PD‐L1 leads to the formation of multinucleated cells and affects sister chromatid cohesion. This effect is due to PD‐L1 compensating for the loss of Sororin, which is suppressed in cancer cells that overexpress PD‐L1. Moreover, PD‐L1 competes with Wing Apart‐Like (WAPL) to bind to PDS5B, ensuring proper sister chromatid cohesion and segregation.

Our gene ontology analysis of nuclear protein fractions from two HNC cell lines showed that PD‐L1 interacts with molecules involved in DNA remodeling and mRNA splicing. This observation aligns with the findings of Yu et al. and supports PD‐L1's role in chromatin remodeling processes in HNC cells [50]. PD‐L1 regulates the DNA damage response by functioning as an RNA binding protein, which regulates many DNA repair proteins. This function highlights PD‐L1 as a potential RNA binding protein that might increase drug resistance in cancer cells [51]. Our analysis also suggests a potential functional relationship of PD‐L1 with the RNA splicing processes. For instance, the genes EIF4A3, HNRNPH2, NONO, CSTF1, and MACROH2A1 are involved in various aspects of RNA metabolism and gene expression regulation. EIF4A3, HNRNPH2, and NONO are particularly involved in pre‐mRNA splicing. All five genes encode proteins that operate within the nucleus, participating in crucial nuclear processes, such as splicing, transcription regulation, and RNA transport, which are essential for proper gene expression and cellular function. All five genes encode proteins that are involved in RNA metabolism to some extent. EIF4A3 is a member of the DEAD‐box protein family and acts as an ATP‐dependent RNA helicase involved in pre‐mRNA splicing as a component of the spliceosome [52]. EIF4A3 is a core component of the exon junction complex, influencing nonsense‐mediated mRNA decay [53]. HNRNPH2 encodes a protein that is part of a family of RNA binding proteins, which act as a shuttle between the nucleus and the cytoplasm, influencing pre‐mRNA splicing. HNRNPH2 acts on pre‐mRNA to affect spliceosome assembly at nearby splice sites [54]. NONO is a DNA‐ and RNA‐binding protein involved in several nuclear processes, including pre‐mRNA splicing, activation, and termination of transcription [55]. NONO is implicated in pre‐mRNA splicing among other gene regulation steps [56]. Moreover, NONO is implicated in activation and termination of transcription, as well as DNA unwinding and repair [57]. CSTF1 is part of the cleavage stimulation factor (CSTF) involved in the polyadenylation and 3′ end cleavage of pre‐mRNAs [58]. MACROH2A1 is a variant of histone H2A and is involved in transcription repression and chromatin structure modulation [59]. MACROH2A1 is known to repress transcription and participate in stable X chromosome inactivation [60].

**Fig. 9:**
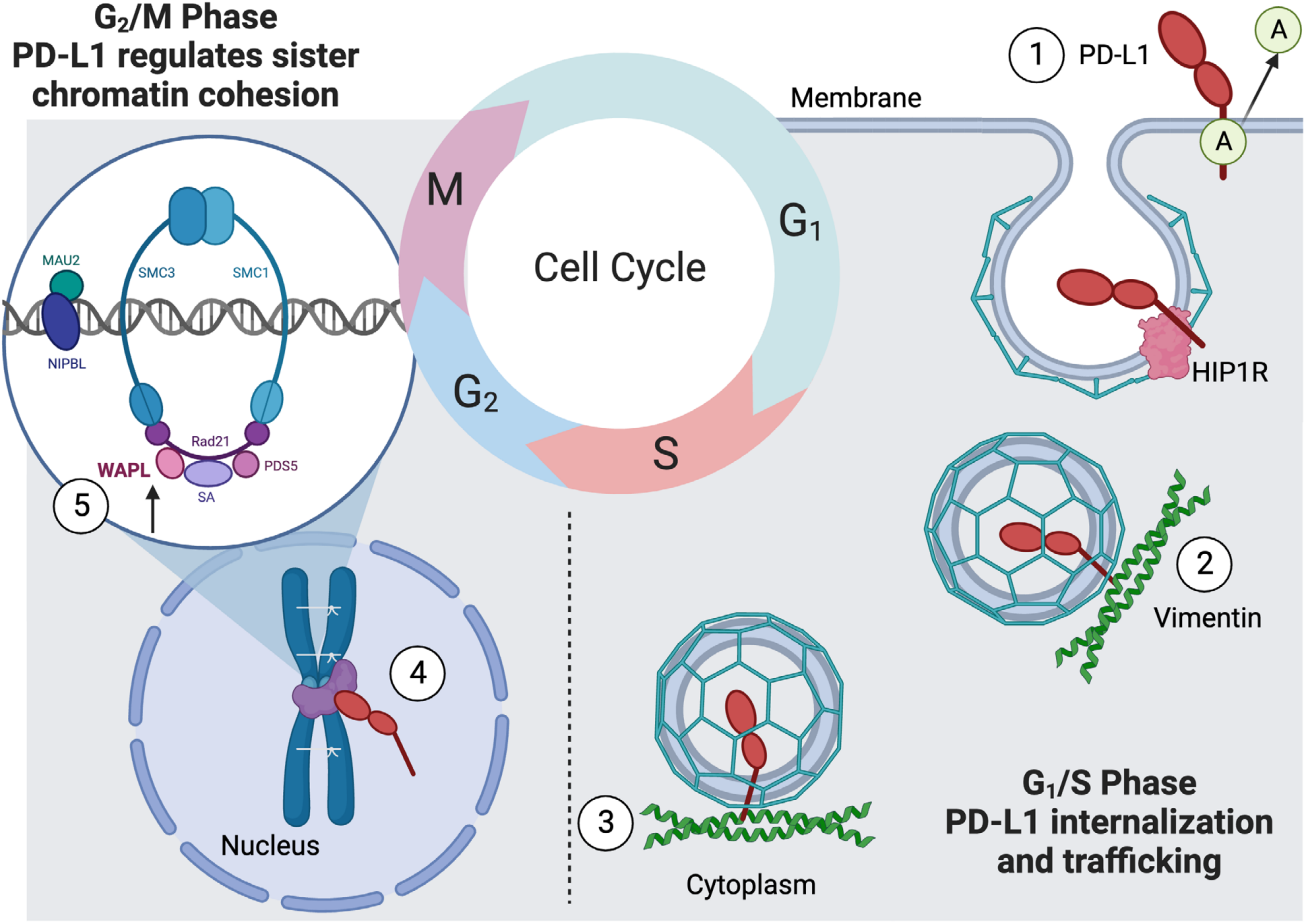
Schematic illustration of the cell‐cycle‐dependent trafficking of PD‐L1 from the membrane to the nucleus via clathrin‐coated pits. The depiction highlights a potential cytoskeletal nucleo‐cytoplasmic shuttle system utilized by PD‐L1 in a cell cycle‐dependent manner, even in the absence of a nuclear localization sequence. The initiation of PD‐L1 shuttling via deacetylation is proposed to be influenced by chemotherapeutic intervention and irradiation treatment. This representation provides insight into why immune checkpoint therapy might have limited efficacy in specific tumor types, such as head and neck tumors or certain brain tumors. Step 1: PD‐L1 membrane interaction with the HIP1R protein during G1 phase [27]. Step 2: PD‐L1 in cytoplasmic trafficking. Illustration of PD‐L1 internalization and trafficking by means of vimentin interaction [27]. Step 3: PD‐L1 travels along endosomal vesicles via vimentin [61]. Steps 4 and 5: PD‐L1 in chromatin regulation. The involvement of PD‐L1 in the G2/M phase within the nucleus, possibly regulating sister chromatid cohesion [28]. Image created using Biorender.

Figure 9 presents a schematic depiction of cell‐cycle‐dependent PD‐L1 trafficking from the cellular membrane to the nucleus. During cell cycle progression PD‐L1 is deacetylized and internalized in clathrin‐coated pits. Interacting with cytoskeletal proteins like vimentin, PD‐L1 utilizes a nucleo‐cytoplasmic shuttle system in a cell cycle‐regulated manner, independent of a nuclear localization signal. This shuttle system's dependence on chemotherapy and irradiation is highly likely, as demonstrated in our previous research. In the nucleus, PD‐L1, for instance, facilitates separation of the sister chromatids during mitosis [28]. These findings may offer insights into why immune checkpoint therapy has limited effectiveness in specific tumor types, such as HNC. Understanding the complex regulation mechanisms of PD‐L1 will further allow antibody‐based immunotherapy to be optimized.

## 5 Conclusion

In summary, our data may provide an insight as to why differential response to PD‐L1 antibody therapy may occur during HNSCC treatment. Detailed understanding of the various immune‐independent tumor cell‐intrinsic PD‐L1 functions, such as the cellular PD‐L1 localization and expression in different cellular compartments during cell cycle progression, will further allow antibody‐based immunotherapy to be optimized. Our results in HNC cells suggest that there is a highly complex regulation of PD‐L1 and that there are several tumor cell‐intrinsic, rather complex functions of PD‐L1 which are independent of immune regulation. These observations may have a major impact on the therapeutic success of approved antibody therapies.

## Conflict of interest

The authors declare no conflict of interest.

## Author contributions

Conceptualization: DS, RJB, TE. Methodology: DS, LF, DSR, SH, SR, YR, MF, MW, GB, AKW, FP. Software: MF, YR, GB, AKW. Validation: DS, RJB, LF, MF and YR, GB, AKW. Formal analysis: DS, MF, YR. Investigation: RJB, TE, DS, LF, DSR, SR, NS, SS. Data curation: RJB, DS, MF, YR. Writing ‐ original draft preparation: DS, RJB, TE. Writing – review and editing: DS, RJB, DSR, EB, NS, SS. Supervision: RJB, DS, JF, EB, ELR, TER. Resources: TE, RJB, TER, JF, FP, SH, YR, EB, ELR, GB, AKW. Project administration: RJB and TE All authors have read and agreed to the published version of the manuscript.

## Supporting information


**Fig. S1.** PD‐L1 localization in subcellular fractions of HNC cell lines and HNSCC tissue, including further cellular characterization.
**Fig. S2.** Validation of subcellular protein fraction purity.
**Fig. S3.** Alternative subcellular protein fractionation method.
**Fig. S4.** Specificity of PD‐L1 immunodetection.
**Fig. S5.** Cell cycle‐dependent expression of nuclear PD‐L1 variants.
**Fig. S6.** Origin of high molecular weight nuclear PD‐L1 variants.
**Fig. S7.** Interacting partners of nuclear PD‐L1.
**Fig. S8.** Cell cycle dependent interaction of PD‐L1 with Vimentin.

## Data Availability

The data that supports the findings of this study are available in the supplementary material section of this article.
