# Supplementary material for: Subcellular localization of PD‐L1 and cell‐cycle‐dependent expression of nuclear PD‐L1 variants: implications for head and neck cancer cell functions and therapeutic efficacy
Source: Mol Oncol. 2023 Dec 26;18(2):431–52. doi: 10.1002/1878-0261.13567 (PMC10850815; doi:10.1002/1878-0261.13567)
Supplement: Supplementary file 1 — Fig. S1. PD‐L1 localization in subcellular fractions of HNC cell lines and HNSCC tissue, including further cellular characterization. Fig. S2. Validation of subcellular protein fraction purity. Fig. S3. Alternative subcellular protein fractionation method. Fig. S4. Specificity of PD‐L1 immunodetection. Fig. S5. Cell cycle‐dependent expression of nuclear PD‐L1 variants. Fig. S6. Origin of high molecular weight nuclear PD‐L1 variants. Fig. S7. Interacting partners of nuclear PD‐L1. Fig. S8. Cell cycle dependent interaction of PD‐L1 with Vimentin. [file MOL2-18-431-s001.zip › mol213567-sup-0001-Supinfo.docx]

**Supplementary Figure S1 PD-L1 localization in subcellular fractions of HNC cell lines and HNSCC tissue, including further cellular characterization.**

Protein fractions include cytoplasmatic (CP), membrane (M), nuclear soluble (NS), nuclear chromatin-bound (NCB), and cytoskeletal (CS) fractions. Each blot shows total protein detection using Ponceau S staining and PD-L1 detection. **A** PD-L1 expression in HNC cell lines A-253, D-562, FaDu, PCI 52, SCC-9 and SCC-15. SCC-9 was chosen as representative cell line in Fig. 2. Consistent PD-L1 expression patterns were observed in the subcellular protein fractions of all analyzed cell lines. Specifically, a PD-L1 band at ~70 kDa was detectable in the NS fraction, while a PD-L1 band >150 kDa was observed in both the NS and NCB fractions. For WB (western blot) analysis, 20 µg of protein was loaded onto a 10% acrylamide gel. **B** Subcellular protein fractionation of seven primary tumors from HNSCC (head and neck squamous cell carcinoma) cancer patients (PT#1, PT#2, PT#3, PT#4, PT#5, PT#7 PT#8). PT#1 were chosen as representative in Fig. 2. In primary tumors, PT#1 and PT#5 prominently exhibited PD-L1 expression at a molecular weight of ~70 kDa and >150 kDa in the nuclear fractions. The ~70 kDa PD-L1 band was also detected in PT#2, PT#3, and PT#4, while the >150 kDa PD-L1 band was observed in PT#3. These findings indicate a consistent expression pattern of PD-L1 across different subcellular protein fractions in both HNC cell lines and HNSCC primary tumors. The presence of PD-L1 variants at specific molecular weights suggests potential regulatory mechanisms and highlights the relevance of subcellular localization in PD-L1 expression in HNC. **C** WB analysis of tumor marker expression in six HNC cell lines A-253, D-562, FaDu, PCI 52, SCC-9 and SCC-15 and **D** six HNSCC tumor patient samples (PT#3 - PT#8). Evaluation included the PD-1/PD-L1 status, PD-L2, EMT markers (Vimentin, E-Cadherin, N-Cadherin), oncogenes (TWIST, Snai1), and the HNC stem cell marker CD44. For WB analysis, 30 µg of protein lysate was loaded onto a 10% acrylamide gel. Expression analysis of β-Actin served as the loading control. **E** Additional information about the origin of the HNC cell lines, including the sex, age of cancer patient, tumor type, and anatomic site. **F** Additional information about the origin of the HNSCC cancer patient samples PT#1 - PT#8, including details about the sex and age of the cancer patient, the anatomic site, and the TNM status of the tumor. Both, HNC cell lines and primary tumors analyzed in this study, exhibited a wide range of cellular characteristics and differentiation status. Interestingly, most cells showed nuclear expression of PD-L1 variants. This finding underscores the significance of PD-L1 nuclear variant expression and its potential involvement in HNC pathogenesis across different cell types.

**Supplementary Figure S2 Validation of subcellular protein fraction purity.**

**A** Original blots for validation of subcellular protein fraction purity in HNC (head and neck) cell line PCI 52. Additional validation of subcellular protein fraction purity in HNC cell line FaDu (**B**) and SCC-9 (**C**). For western blot analysis, 20 µg of protein was loaded onto a 10% acrylamide gel. Markers for specific subcellular compartments were detected to confirm the accuracy of the subcellular fractionation technique, ensuring minimal cross-contamination, and emphasizing the reliability of this method in accurately characterizing subcellular protein localization. Cytoplasmic markers: GAPDH and Calpain-2, membrane markers: Na-, K-, ATPase, and EGFR, nucleic markers: Histone H3 and Lamin A/C, cytoskeletal markers: β-Actin and β-Tubulin, total protein staining: Ponceau S.

**Supplementary Figure S3 Alternative subcellular protein fractionation method**.

In this set of experiments, we utilized an alternative subcellular protein fractionation technique following Abcam's 'Subcellular Fractionation Protocol.' Unlike the 'Subcellular Fractionation Kit' from Thermo Scientific, which employs separate buffers and centrifugation steps for fractionation, this approach employs a uniform buffer with distinct centrifugation steps to segregate cells into cytoplasmic (C), membrane (M), and nuclear protein fractions (N). We examined PD-L1 expression in head and neck (HNC) cell lines A-253, D-562, FaDu, PCI 52, SCC-9, and SCC-15. Like the methodology employed with the 'Subcellular Fractionation Kit' from Thermo Scientific, the analysis revealed varying PD-L1 molecular weights depending on the specific cellular fraction. Notably, the nuclear fractions exhibited unique PD-L1 variants at ~70 kDa and >150 kDa. To validate the purity of the fractions, we used HNC cell line PCI 52. Specific marker proteins, including cytoplasmic markers GAPDH and Calpain-2, membrane markers Na-, K-, ATPase, and EGFR, nucleic markers Histone H3 and Lamin A/C, along with cytoskeletal markers β-Actin and β-Tubulin, were assessed for their respective subcellular localizations. Ponceau S staining is used as a total protein marker. For each analysis, 20 µg of protein was loaded onto a 10% acrylamide gel. Expression patterns of these markers varied based on the specific fraction being examined. The results confirmed purity of the cellular protein fractions, with minimal cross-contamination between the fractions. This alternative subcellular fractionation method provides additional evidence of distinct PD-L1 variants.

**Supplementary Figure S4 Specificity of PD-L1 immunodetection.**

Head and neck cancer (HNC) cell lines FaDu and PCI 52 were used for PD-L1 siRNA knockdown (KD) experiments, with transfection of non-targeting (NT) siRNA serving as the control. Controls were harvested at 2, 5, and 8 days after transfection (lanes 1-3), while cells transfected with PD-L1 siRNA were harvested from 1 to 8 days after transfection (lanes 4-11). Cells were seeded with appropriate cell numbers during days 1 to 4. For samples analyzed from day 5 to 8, a passage on day 4 was conducted to ensure consistent confluence levels, optimal nutrition, and reduced selective pressure. Adjustments in the number of cells per area were made to maintain similar confluence levels by seeding more densely for early harvesting time points and using fewer cells for late harvesting time points. For Western Blot analysis, 20 µg of protein was loaded onto 10% acrylamide gels. Each blot presented in this figure represents either the total protein or specific cellular fractions obtained through cellular fractionation, including the cytoplasmic (CP), membrane (M), nuclear soluble (NS), nuclear chromatin-bound (NCB), and cytoskeletal (CS) protein fractions. Housekeeping proteins were detected as loading controls, with β-Actin chosen for total protein lysate and cytoskeletal fractions, GAPDH for cytoplasmic fraction, Na-, K-, ATPase for membrane fraction, Lamin A/C for NS fraction, and Histone H3 for NCB fraction. The time-dependent decrease in signal detection observed in this study provides evidence for the specificity of the PD-L1 antibody used. Interestingly, a delay in signal decrease was observed, which varied depending on the cellular fraction and PD-L1 variant. This delay can be explained by the differential stabilization of PD-L1, depending on the specific cellular fraction (62). Furthermore, the observed increase in signal at late harvesting time-points can be attributed to the performance of transient KD, indicating the transient nature of the PD-L1 KD.

**Supplementary figure S5 Cell cycle-dependent expression of nuclear PD-L1 variants.**

Original blots with subcellular fractionations after cell cycle inhibition of head and neck (HNC) cell line FaDu and PCI 52. **A** Each blot represents the specific subcellular fractions of samples treated with palbociclib (G1), aphidicolin (S) or nocodazole (G2/M). Total protein (Ponceau S staining) and PD-L1 expression was detected. **B** Each blot represents a subcellular fraction, including the cytoplasmic (CP), membrane (M), nuclear soluble (NS), nuclear chromatin-bound (NCB), and cytoskeletal protein (CS) of samples treated with the specific cell cycle inhibitors. Additionally, control samples treated with DMSO, resulting in a random distribution of cells in any cell cycle phase, were included. For western blot analysis, 20 µg of protein was loaded onto a 10% acrylamide gel. The analysis revealed the specific expression of high molecular weight PD-L1 variants exclusively in the nuclear fractions. Moreover, the expression of these variants was found to be dependent on the state of cell cycle progression. These findings highlight the cell cycle-regulated expression of PD-L1 variants and suggest their potential involvement in cell cycle-related processes. **C** Immunofluorescent staining of PD-L1 in synchronized HNC cells from PCI 52 at different cell cycle phases. Displayed are the images from Fig. 4 with a highlighted nuclear border. **D** Images from Fig. 4 only with PD-L1 staining (green). Magnification = 40x, scale bar = 50 µm.

**Supplementary figure S6 Origin of high molecular weight nuclear PD-L1 variants.**

Two approaches were employed: excessive protein heating to provoke protein dissociation, and deglycosylation. For these experiments, 20 µg of nuclear soluble (NS) or chromatin-bound (NCB) protein from head and neck (HNC) cell line FaDu was used. Proteins were separated in a 4-12% gradient gel. **A** Samples were subjected to heating at either 70°C (lanes 1-4) or 95°C (lanes 5-8) for 5, 10, 30, or 60 min. **B** Deglycosylation of proteins, achieved by digesting with either PNGase F (lanes 1-4) or Endo H (lanes 5-8). Control samples are indicated as (-), enzymatically digested samples are indicated as (+). Lamin A/C was used as housekeeping protein. The results revealed that excessive heating of nuclear samples led to a molecular weight shift of PD-L1 from >150 kDa to ~70 kDa. Digestion with PNGase F only resulted in a molecular weight shift of the 40 - 55 kDa PD-L1 to ~34 kDa. Endo H exhibited a similar but weaker effect. These findings indicate that the high molecular weight PD-L1 is likely a result of PD-L1 multimerization. Noteworthy, the >150 kDa PD-L1 signal increases after digestion of glycosylation, which can be explained by an increased accessibility of antibody binding sites after removal of glycosylation.

**Supplementary figure S7 Interacting partners of nuclear PD-L1.**

**A** For mass spectrometry analysis 100 µg nuclear protein fraction from head and neck (HNC) cell line FaDu and PCI 52 was used to capture PD-L1 in co-immunoprecipitation (CoIP). An immunoglobulin G (IgG) isotype control antibody (Iso ab) served as control for unspecific binding during CoIP with the monoclonal anti-PD-L1 IgG capture antibody (PD-L1 ab). WB after CoIP indicates specificity of PD-L1 capture antibody for Co-IP. Gel was loaded with 5 µl from 50 µl CoIP eluate. **B** List of PD-L1 interacting partners found in mass spectrometry analysis of nuclear lysates from HNC cell line FaDu and PCI 52. Proteins, captured with IgG isotype control antibody were considered unspecific and have been excluded. Besides proteins related to DNA remodeling and RNA splicing the list reveals specific interaction of PD-L1 with cytoskeletal proteins, like keratins.

**Supplementary figure S8 Cell cycle dependent interaction of PD-L1 with Vimentin.**

**A** Proximity ligation assay (PLA) in head and neck cancer (HNC) cell line PCI 52 was performed to visualize the interaction of PD-L1 with Vimentin as red dots. Cells are synchronized in G1, S or G2/M phase. Nuclei are stained with DAPI (blue), cytoskeleton with Phalloidin (green). With focus on the PD-L1/Vimentin interaction, images in the upper row are displayed without the cytoskeleton staining. For determination of PD-L1 localization, triple staining was performed in the lower row. A respective IgG isotype antibody was used as control. The DMSO control group showed cells distributed randomly across cell cycle phases. All pictures include a highlighted nuclear border. Magnification = 40x, scale bar = 50 µm. **B** Quantification of PD-L1/Vimentin interactions. Single cells were analyzed separately. For every cell, the fluorescence intensity of each PLA signal (red) was measured by Fiji ImageJ software. The corrected total intensity was calculated by subtracting the mean background in relation to the area analyzed (CTCF).
