# Supplementary figures and images for: Subcellular localization of PD‐L1 and cell‐cycle‐dependent expression of nuclear PD‐L1 variants: implications for head and neck cancer cell functions and therapeutic efficacy
Source: Mol Oncol. 2023 Dec 26;18(2):431–52. doi: 10.1002/1878-0261.13567 (PMC10850815; doi:10.1002/1878-0261.13567)

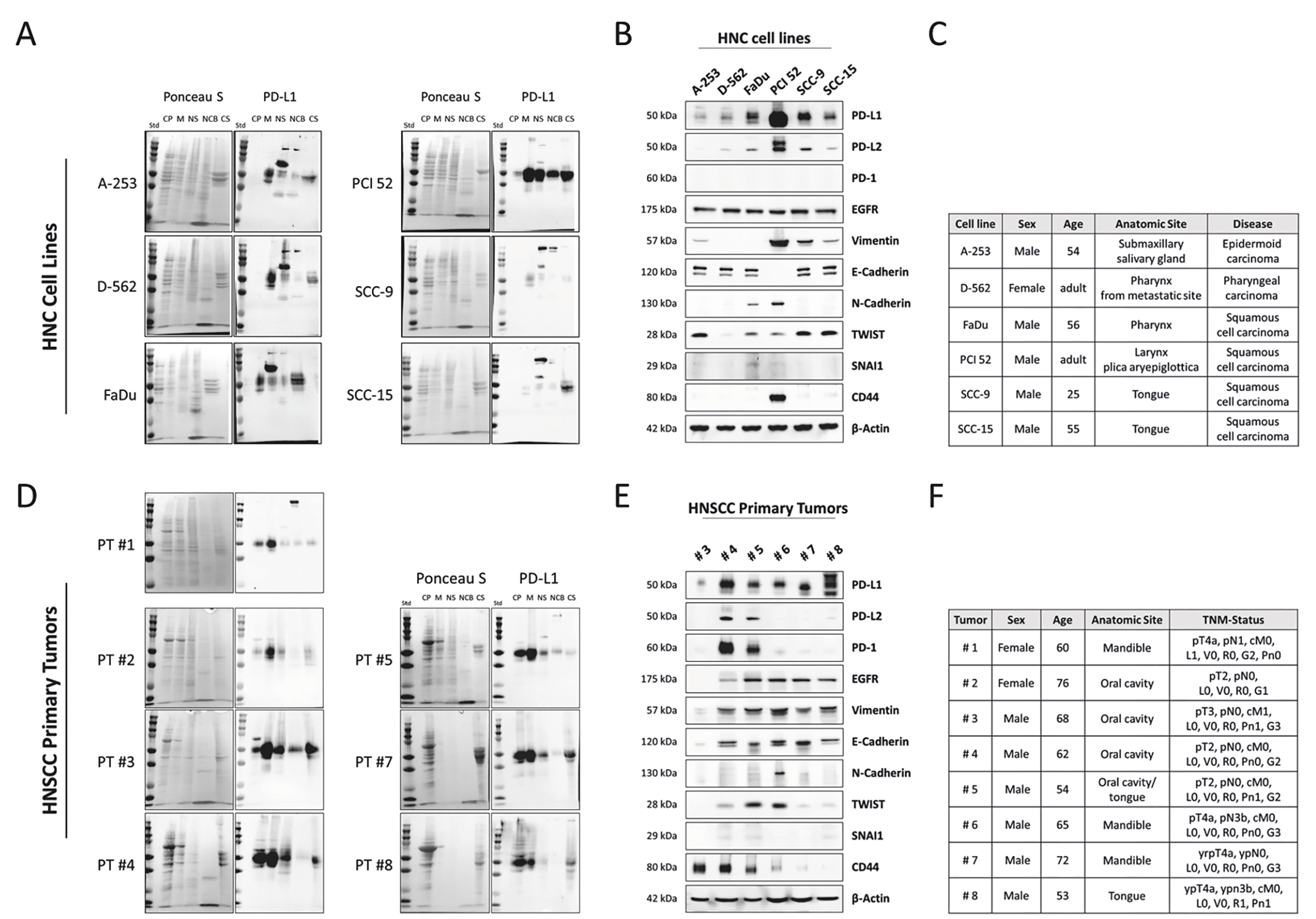

Supplement: Supplementary file 1 — Fig. S1. PD‐L1 localization in subcellular fractions of HNC cell lines and HNSCC tissue, including further cellular characterization. Fig. S2. Validation of subcellular protein fraction purity. Fig. S3. Alternative subcellular protein fractionation method. Fig. S4. Specificity of PD‐L1 immunodetection. Fig. S5. Cell cycle‐dependent expression of nuclear PD‐L1 variants. Fig. S6. Origin of high molecular weight nuclear PD‐L1 variants. Fig. S7. Interacting partners of nuclear PD‐L1. Fig. S8. Cell cycle dependent interaction of PD‐L1 with Vimentin. [file MOL2-18-431-s001.zip › mol213567-sup-0002-FigureS1.tif]

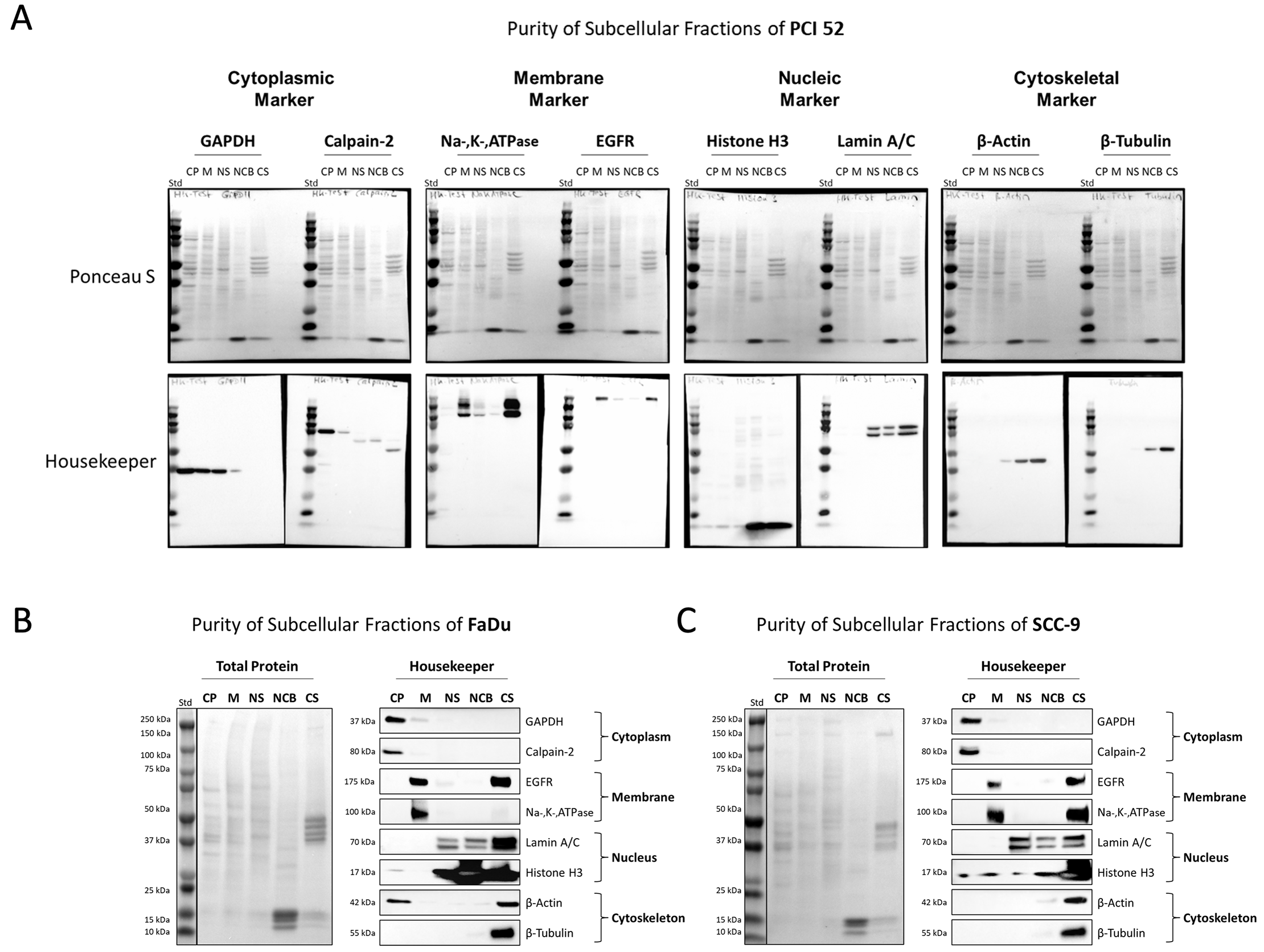

Supplement: Supplementary file 1 — Fig. S1. PD‐L1 localization in subcellular fractions of HNC cell lines and HNSCC tissue, including further cellular characterization. Fig. S2. Validation of subcellular protein fraction purity. Fig. S3. Alternative subcellular protein fractionation method. Fig. S4. Specificity of PD‐L1 immunodetection. Fig. S5. Cell cycle‐dependent expression of nuclear PD‐L1 variants. Fig. S6. Origin of high molecular weight nuclear PD‐L1 variants. Fig. S7. Interacting partners of nuclear PD‐L1. Fig. S8. Cell cycle dependent interaction of PD‐L1 with Vimentin. [file MOL2-18-431-s001.zip › mol213567-sup-0003-FigureS2.tif]

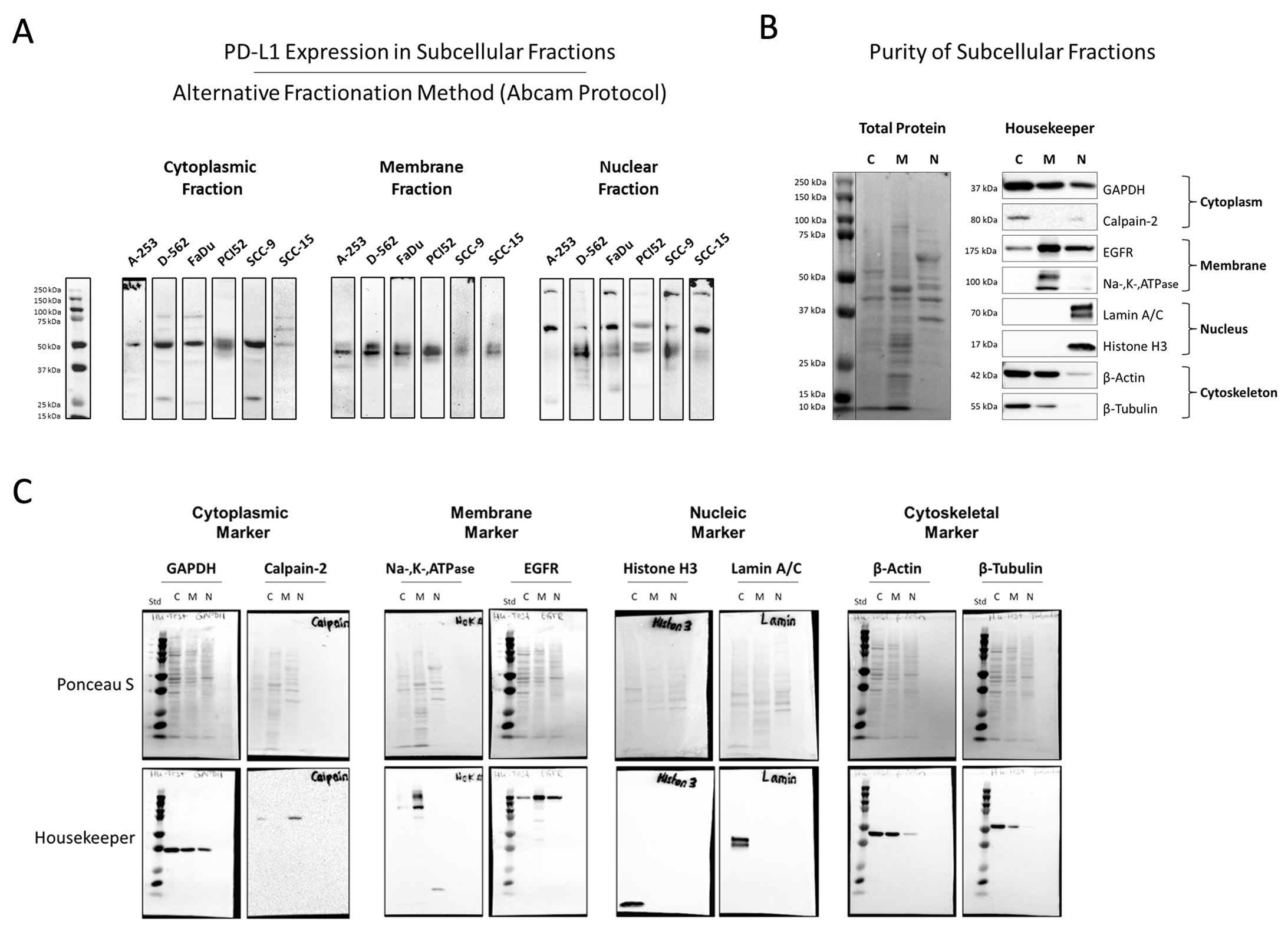

Supplement: Supplementary file 1 — Fig. S1. PD‐L1 localization in subcellular fractions of HNC cell lines and HNSCC tissue, including further cellular characterization. Fig. S2. Validation of subcellular protein fraction purity. Fig. S3. Alternative subcellular protein fractionation method. Fig. S4. Specificity of PD‐L1 immunodetection. Fig. S5. Cell cycle‐dependent expression of nuclear PD‐L1 variants. Fig. S6. Origin of high molecular weight nuclear PD‐L1 variants. Fig. S7. Interacting partners of nuclear PD‐L1. Fig. S8. Cell cycle dependent interaction of PD‐L1 with Vimentin. [file MOL2-18-431-s001.zip › mol213567-sup-0004-FigureS3.tif]

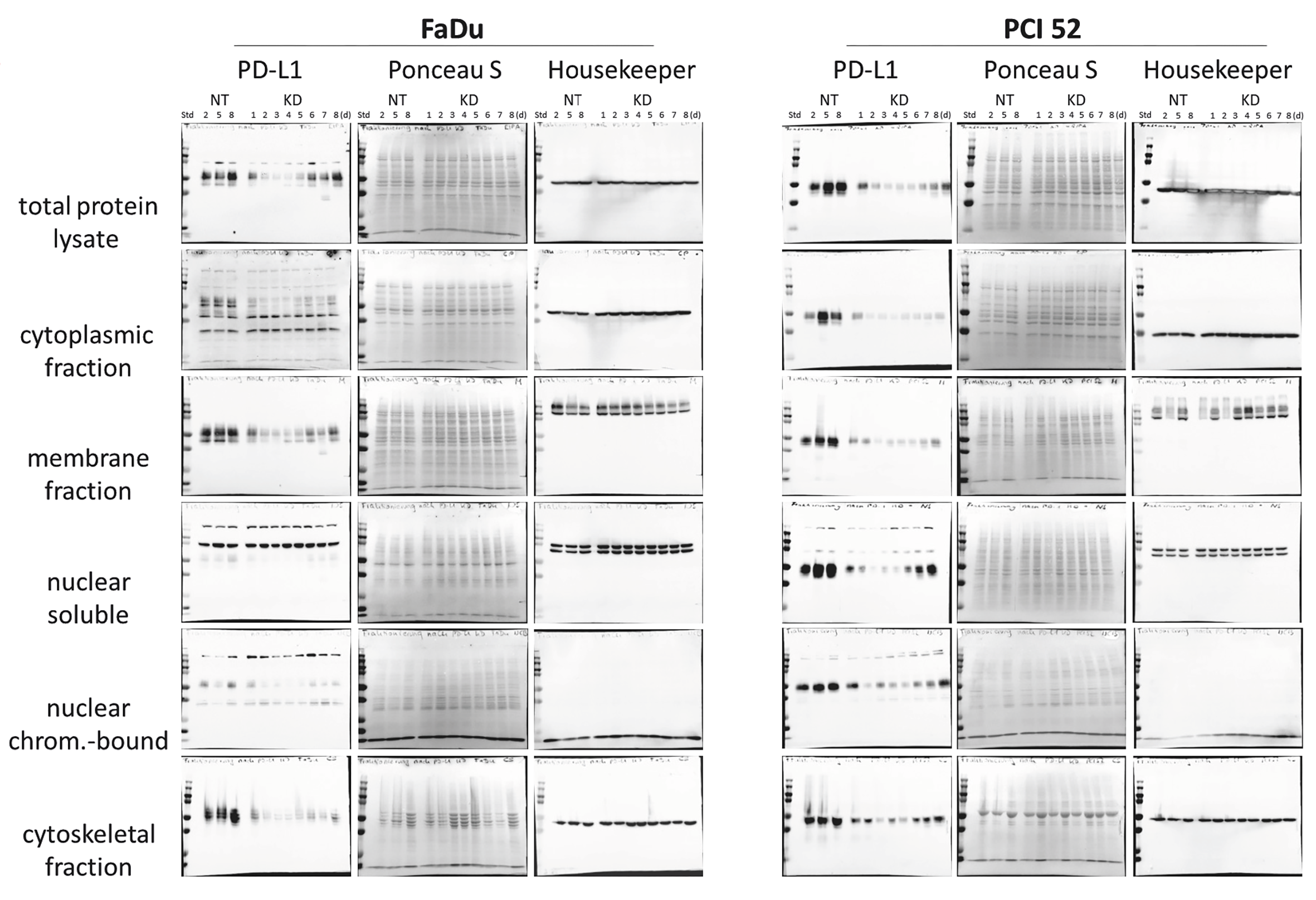

Supplement: Supplementary file 1 — Fig. S1. PD‐L1 localization in subcellular fractions of HNC cell lines and HNSCC tissue, including further cellular characterization. Fig. S2. Validation of subcellular protein fraction purity. Fig. S3. Alternative subcellular protein fractionation method. Fig. S4. Specificity of PD‐L1 immunodetection. Fig. S5. Cell cycle‐dependent expression of nuclear PD‐L1 variants. Fig. S6. Origin of high molecular weight nuclear PD‐L1 variants. Fig. S7. Interacting partners of nuclear PD‐L1. Fig. S8. Cell cycle dependent interaction of PD‐L1 with Vimentin. [file MOL2-18-431-s001.zip › mol213567-sup-0005-FigureS4.tif]

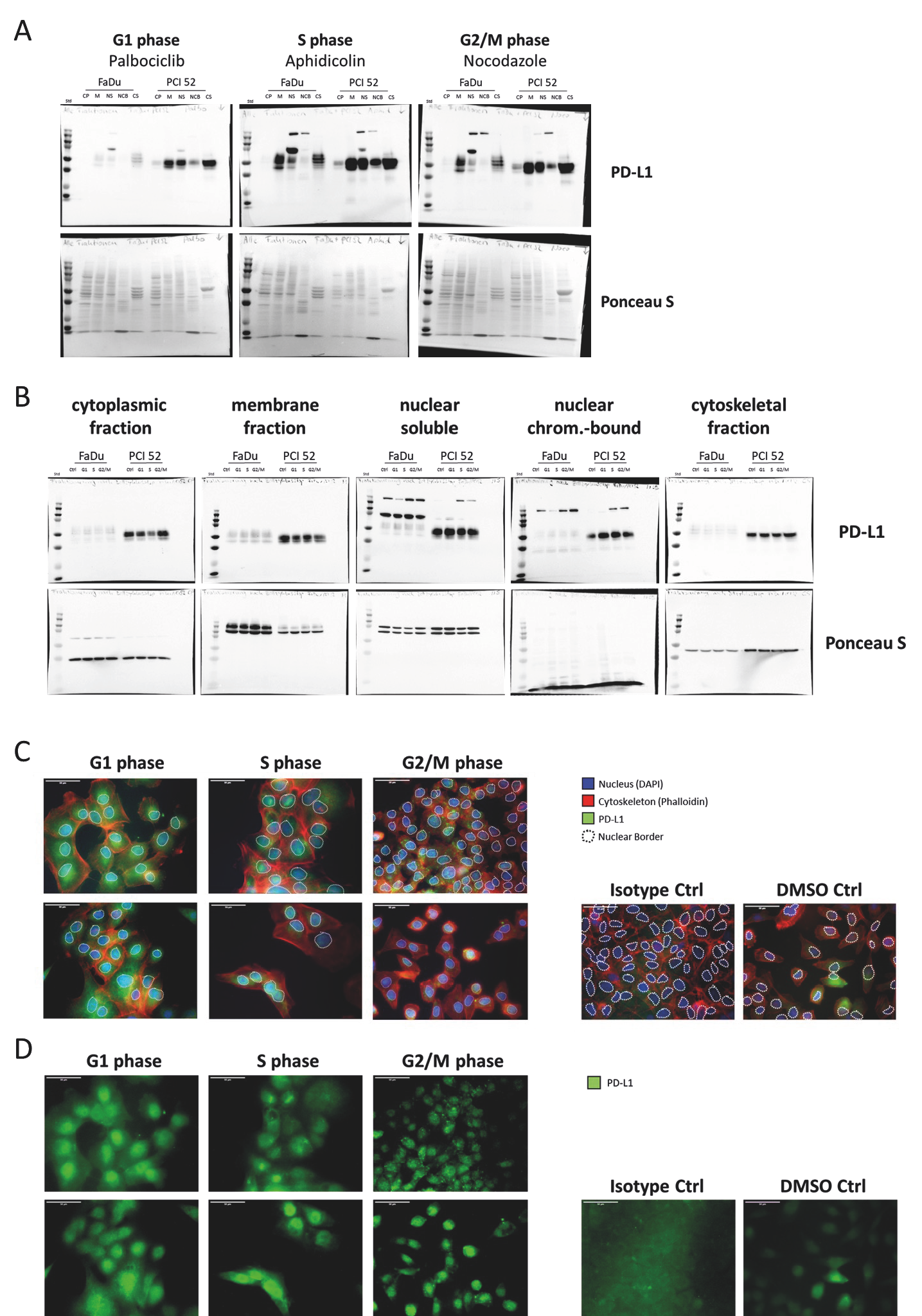

Supplement: Supplementary file 1 — Fig. S1. PD‐L1 localization in subcellular fractions of HNC cell lines and HNSCC tissue, including further cellular characterization. Fig. S2. Validation of subcellular protein fraction purity. Fig. S3. Alternative subcellular protein fractionation method. Fig. S4. Specificity of PD‐L1 immunodetection. Fig. S5. Cell cycle‐dependent expression of nuclear PD‐L1 variants. Fig. S6. Origin of high molecular weight nuclear PD‐L1 variants. Fig. S7. Interacting partners of nuclear PD‐L1. Fig. S8. Cell cycle dependent interaction of PD‐L1 with Vimentin. [file MOL2-18-431-s001.zip › mol213567-sup-0006-FigureS5.tif]

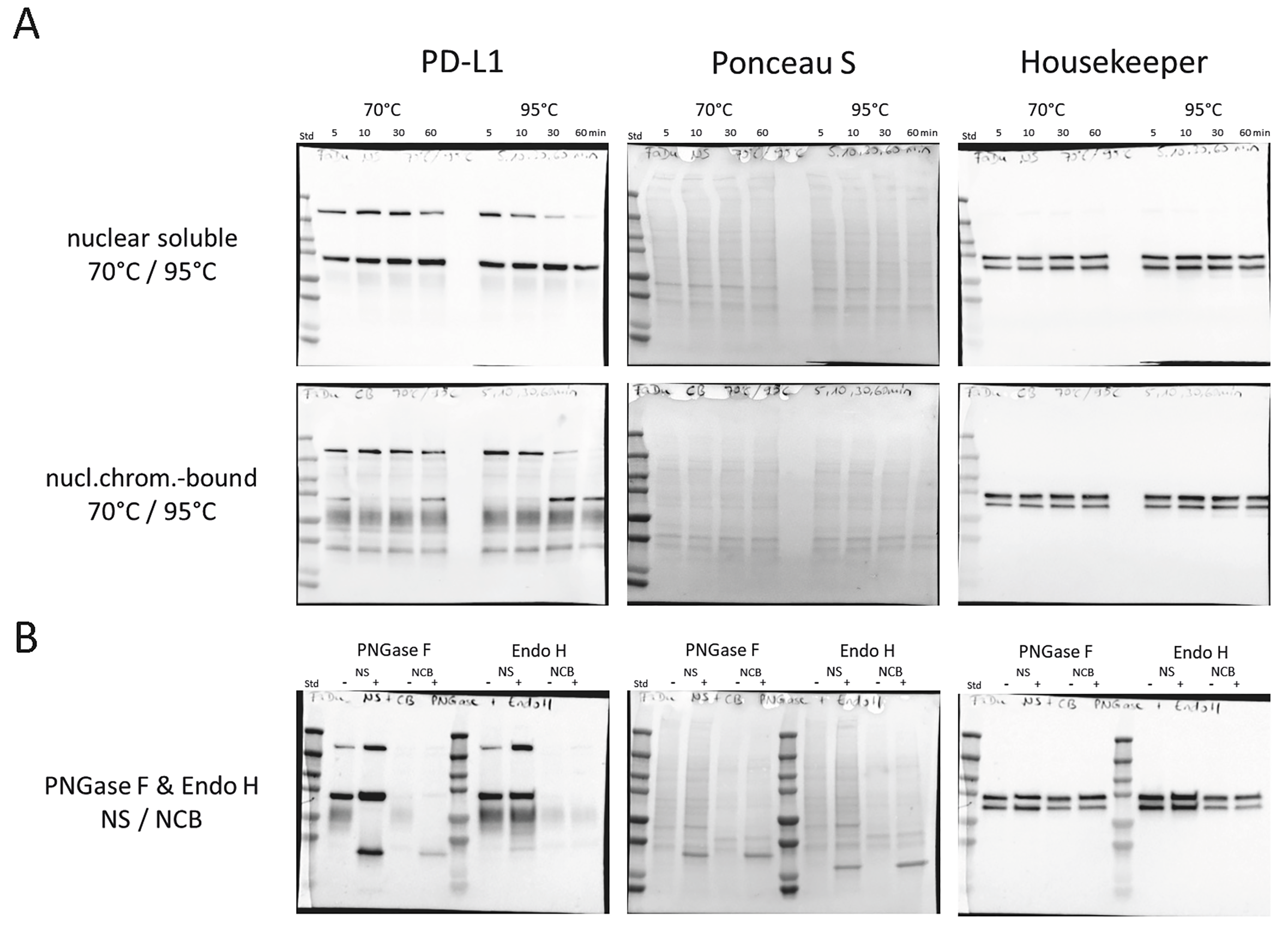

Supplement: Supplementary file 1 — Fig. S1. PD‐L1 localization in subcellular fractions of HNC cell lines and HNSCC tissue, including further cellular characterization. Fig. S2. Validation of subcellular protein fraction purity. Fig. S3. Alternative subcellular protein fractionation method. Fig. S4. Specificity of PD‐L1 immunodetection. Fig. S5. Cell cycle‐dependent expression of nuclear PD‐L1 variants. Fig. S6. Origin of high molecular weight nuclear PD‐L1 variants. Fig. S7. Interacting partners of nuclear PD‐L1. Fig. S8. Cell cycle dependent interaction of PD‐L1 with Vimentin. [file MOL2-18-431-s001.zip › mol213567-sup-0007-FigureS6.tif]

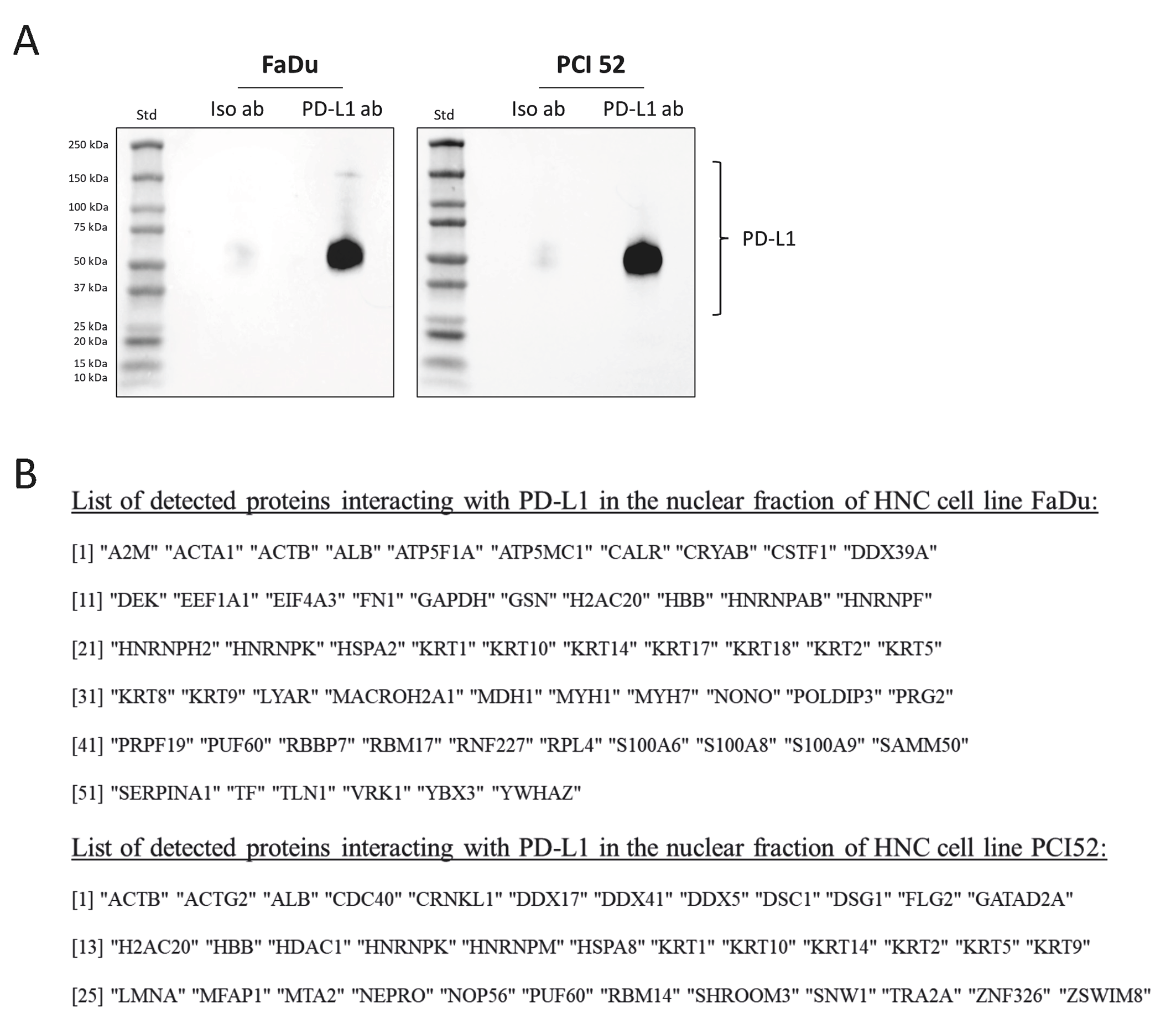

Supplement: Supplementary file 1 — Fig. S1. PD‐L1 localization in subcellular fractions of HNC cell lines and HNSCC tissue, including further cellular characterization. Fig. S2. Validation of subcellular protein fraction purity. Fig. S3. Alternative subcellular protein fractionation method. Fig. S4. Specificity of PD‐L1 immunodetection. Fig. S5. Cell cycle‐dependent expression of nuclear PD‐L1 variants. Fig. S6. Origin of high molecular weight nuclear PD‐L1 variants. Fig. S7. Interacting partners of nuclear PD‐L1. Fig. S8. Cell cycle dependent interaction of PD‐L1 with Vimentin. [file MOL2-18-431-s001.zip › mol213567-sup-0008-FigureS7.tif]

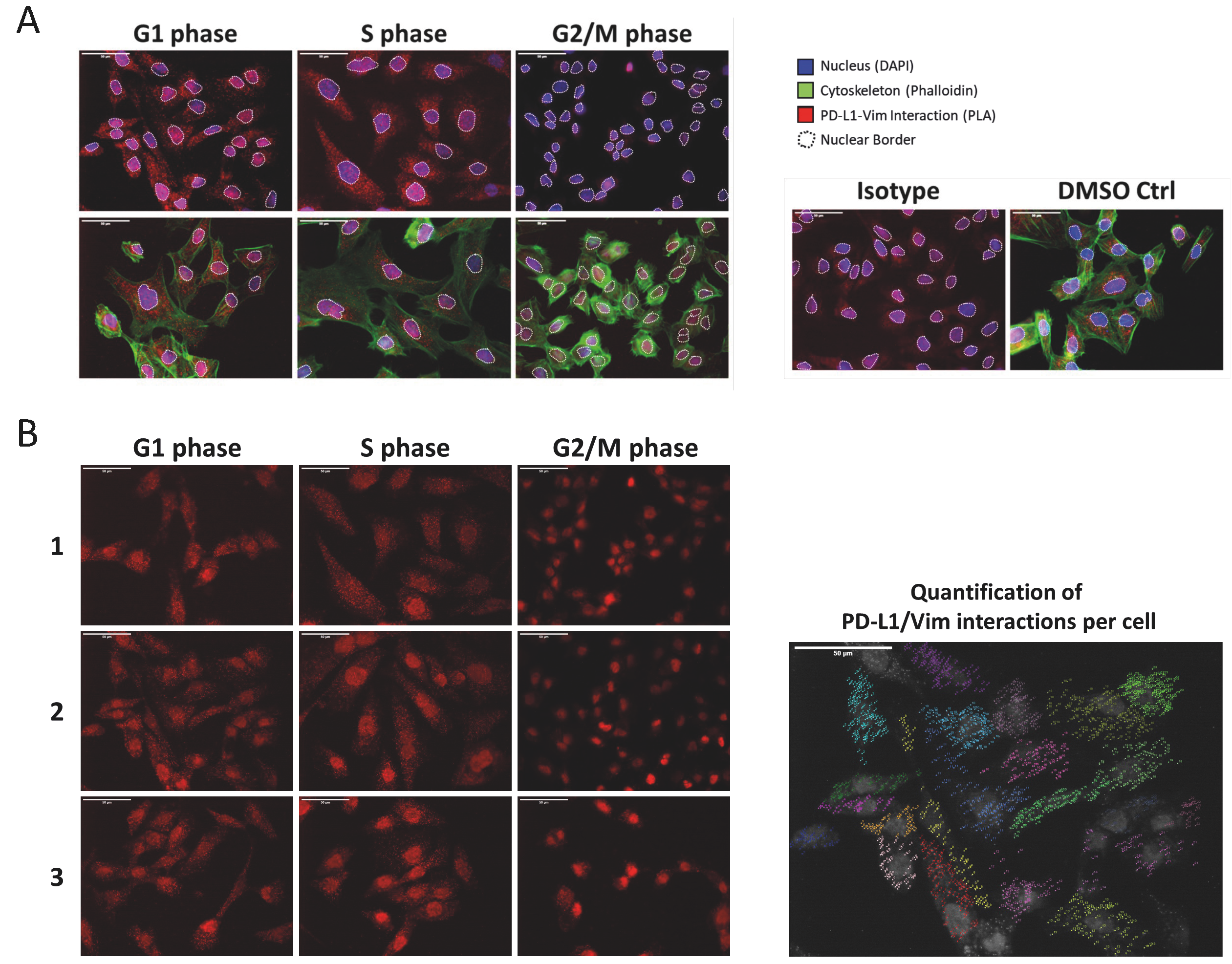

Supplement: Supplementary file 1 — Fig. S1. PD‐L1 localization in subcellular fractions of HNC cell lines and HNSCC tissue, including further cellular characterization. Fig. S2. Validation of subcellular protein fraction purity. Fig. S3. Alternative subcellular protein fractionation method. Fig. S4. Specificity of PD‐L1 immunodetection. Fig. S5. Cell cycle‐dependent expression of nuclear PD‐L1 variants. Fig. S6. Origin of high molecular weight nuclear PD‐L1 variants. Fig. S7. Interacting partners of nuclear PD‐L1. Fig. S8. Cell cycle dependent interaction of PD‐L1 with Vimentin. [file MOL2-18-431-s001.zip › mol213567-sup-0009-FigureS8.tif]
